# A Review of Global Optimization Methods for Molecular Structures: Algorithms, Applications and Perspectives

**DOI:** 10.1002/jcc.70243

**Published:** 2025-10-20

**Authors:** Jorge Alberto Sanchez Alvarez, Patrizia Calaminici

**Affiliations:** ^1^ Chemistry Department CINVESTAV Mexico City Mexico

**Keywords:** algorithms for exploring potential energy surfaces, deterministic method, global optimization methods, molecular structures, stochastic method

## Abstract

This review presents a comprehensive overview of global optimization techniques applied to the prediction of chemical structures, including molecular conformations, crystal polymorphs, and reaction pathways. These approaches typically involve a two‐step process: a global search to identify candidate structures, followed by local refinement to determine the most stable configurations. Global optimization methods are commonly grouped into two categories, known as stochastic and deterministic methods, based on their exploration strategies and underlying theoretical principles. A historical perspective highlights the progression of these methods, from early foundational algorithms to more advanced and efficient modern techniques. For each category, key algorithmic frameworks are outlined, widely used software tools are discussed, and representative applications are examined, such as conformer sampling, cluster structure prediction, and surface adsorption. The review concludes by considering future directions, including the integration of accurate quantum methods, the development of flexible hybrid algorithms, and the use of quantum computing to address increasingly complex chemical problems.

## Graphical abstract

This image categorizes global optimization methods into stochastic and deterministic approaches. Stochastic methods include techniques like Genetic Algorithms, Simulated Annealing, and Machine Learning, often involving randomness. Deterministic methods, such as Molecular Dynamics and Single‐Ended methods, follow defined rules without randomness. Each method is illustrated with icons, and the diagram visually distinguishes the two categories for clear scientific presentation.

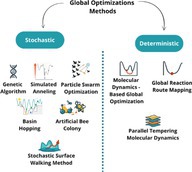

## 1 Introduction

Global optimization (GO) plays a central role in modern computational science, particularly in predicting of molecular and material structures. It involves locating the most stable configuration of a system, defined as the geometry corresponding to the lowest point on its potential energy surface (PES). In molecular systems, this global minimum (GM) is essential for accurately predicting a wide range of properties, including thermodynamic stability, reactivity, spectroscopic behavior, and biological activity [1, 2]. These predictions are critical in fields such as drug discovery, catalysis, and materials design. In recent years, first‐principles density functional theory (DFT) [3, 4, 5] methods have been widely adopted for their favorable balance between accuracy and computational cost. Among these, auxiliary density functional theory (ADFT) [6, 7], a low‐scaling variant of Kohn‐Sham DFT, is particularly well suited for large and complex systems [8]. Furthermore, ADFT provides numerically stable first‐ and second‐order analytic derivatives essential for efficient PES exploration. Numerous GO algorithms have been developed to address the computational challenges associated with accurate structure prediction [9, 10, 11]. A central concept in molecular structure prediction lies the concept of the PES. The PES is a multidimensional hypersurface that maps the potential energy of a molecular system as a function of its nuclear coordinates. Each point on the PES corresponds to a specific molecular geometry, and the topological features of the surface, including its minima, saddle points, and maxima, provide essential insights into molecular stability and reactivity. Local minima represent energetically stable structures. First‐order saddle points correspond to transition states, typically identified by a single imaginary vibrational frequency that signals a direction of negative curvature. Higher‐order saddle points, which involve multiple such directions, are generally not directly relevant to chemical transformations [12, 13].

**FIGURE 1:**
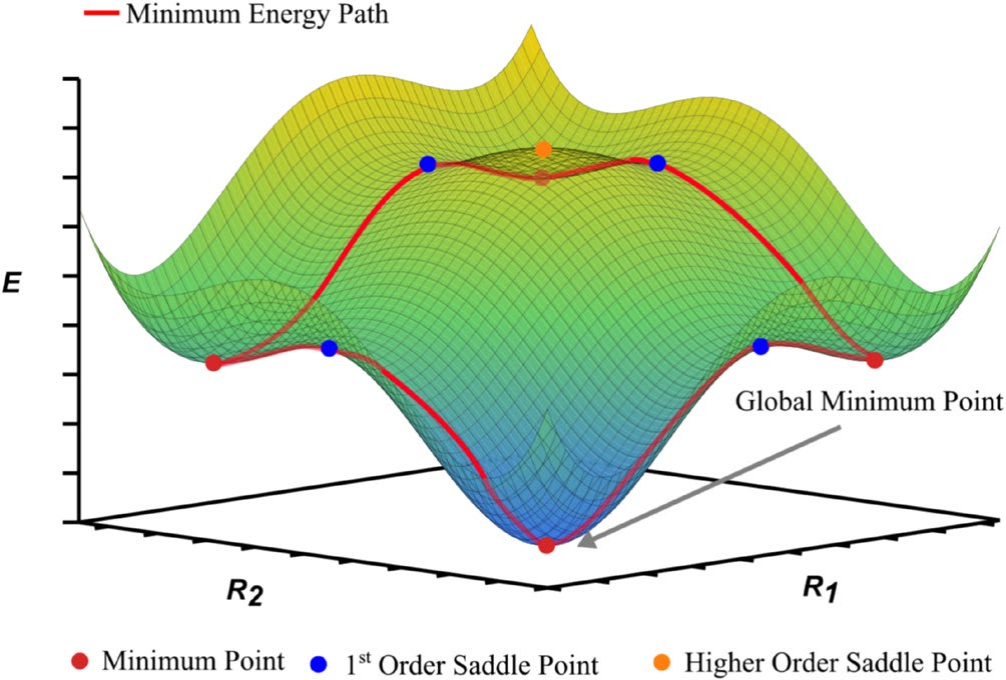
Schematic representation of a potential energy surface (PES) of a system with two nuclear coordinates R_1_ and R_2_. In this figure an example of a PES with local minima, global minimum, transition structure, higher order saddle point and minimum energy path (red line), is illustrated.

A schematic PES is shown in Figure 1, where energy is plotted against two collective coordinates, $R_{1}$ and $R_{2}$. Red spheres mark local minima, which may represent isomers, reactants, or products. These are connected by a red path illustrating the minimum energy route between configurations, known as the minimum energy path (MEP). Blue spheres indicate first‐order saddle points that define energy barriers between local minima, while an orange sphere marks a higher‐order saddle point. The GM, introduced by a gray arrow in this figure, represents the most thermodynamically stable structure. While illustrative, this simplified representation masks the complexity of real PESs, which span high‐dimensional spaces and exhibit a rapidly growing number of local minima as system size increases. Theoretical models suggest that the number of minima scales exponentially with the number of atoms, following a relation of the form $N_{\min}(N)=\exp(\xi N)$, where ξ is a system‐dependent constant [14, 15]. A similar scaling applies to transition states, under the assumption of localized atomic rearrangements. As a result, the energy landscape becomes increasingly complex for larger systems, that is, atomic clusters [16], presenting a significant challenge to global structure prediction. In contrast, local optimization methods are not intended to locate the GM of a system. Instead, they are primarily designed to identify local minima, transition states, and MEPs, focusing on the local topology of the PES near an initial geometry [17]. While highly effective for studying specific reaction mechanisms or refining known structures, these methods are inherently limited in their ability to explore the PES globally.

Many GO algorithms combine global exploration with local refinement. In some cases, these are organized as separate phases, while in others they are intertwined within a single, continuous search process [18]. This approach increases the likelihood of locating low‐energy configurations that are inaccessible to purely local methods. GO methods have proven to be effective across a wide range of chemical systems, including atomic and molecular clusters [19], biomolecules [20], solid‐state materials [21], and drug‐like compounds [22]. Although GO algorithms share a general framework, their implementation varies considerably. A typical workflow begins with the generation of an initial population of candidate structures using techniques such as random sampling, physically motivated perturbations, or heuristic design. Each structure is locally optimized to identify the nearest stationary point, and redundant or symmetrically equivalent structures are removed. Frequency analysis is then used to confirm that each candidate corresponds to a true minimum. Among the remaining structures, the one with the lowest energy is designated as the putative GM. Despite following similar steps, algorithms differ in how they navigate the PES. Some rely on stochastic strategies, while others are inspired by physical models or evolutionary dynamics. Effective algorithms must balance exploration of the surface with exploitation of promising regions, which remains an enduring challenge in the design of GO techniques [23]. Building on this theoretical foundation, the next section introduces the main classes of GO methods. We present a historical overview of their development, classify them into stochastic and deterministic categories, and examine their strategies for navigating complex energy landscapes. In addition, we have selected ten representative studies for each GO method to provide to the readers a brief but meaningful overview of how each algorithm has been applied in chemistry and materials science. These examples were chosen to highlight the diversity, evolution, and impact of each method.

## 2 Global Optimization Methods

**FIGURE 2:**
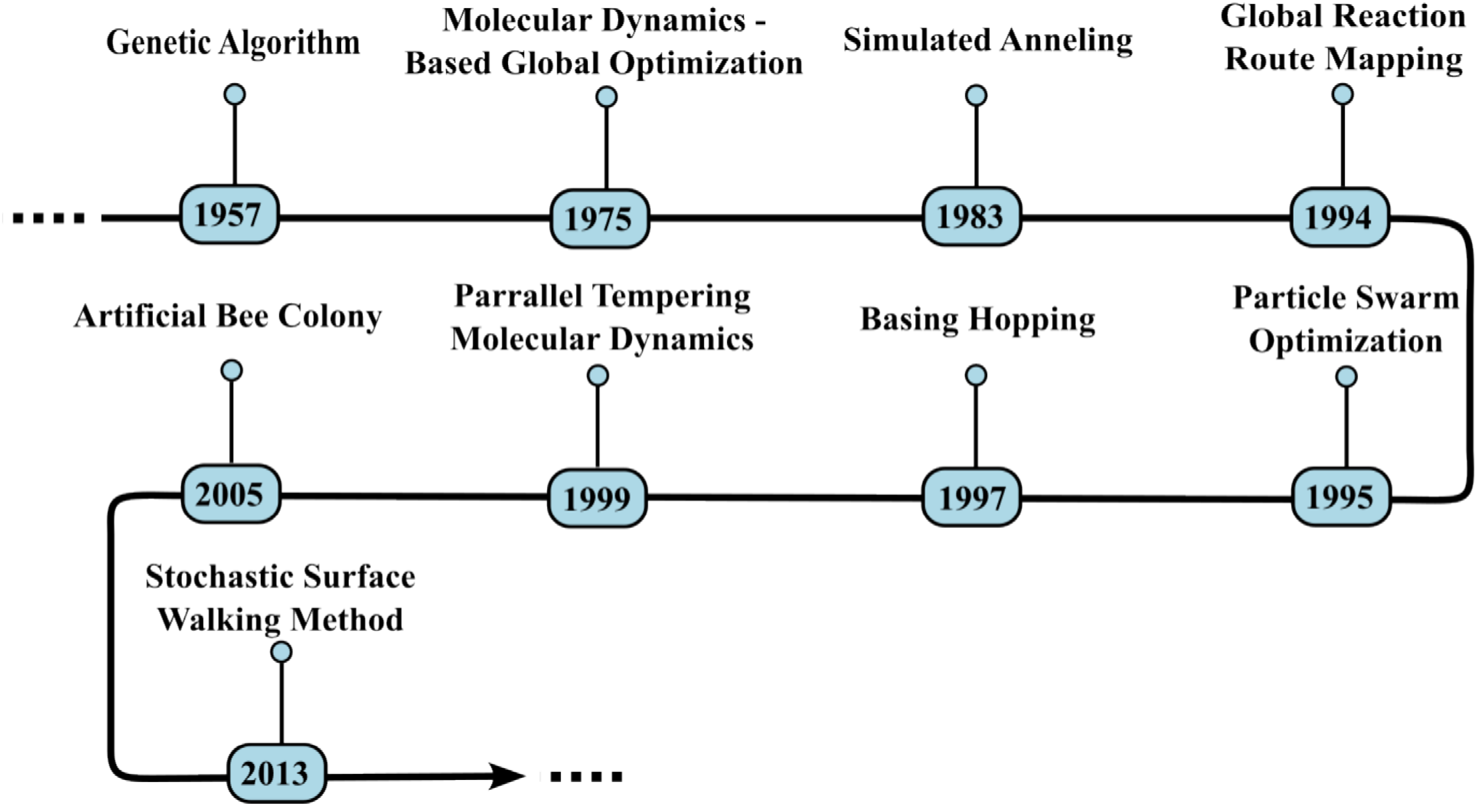
Timeline illustrating significant developments in GO methods for exploring molecular potential energy surfaces.

Over the past several decades, a wide range of GO techniques has been developed to address the challenge of locating the GM on complex PESs. These methods are critical for accurate molecular and material structure prediction, particularly in systems characterized by high dimensionality and complex energy landscapes. A timeline of major developments is presented in Figure 2, highlighting key advances that have shaped the field.

The earliest approach to GO was the population‐based genetic algorithm (GA), formalized in 1957, that applied evolutionary strategies such as selection, crossover, and mutation to optimize structural populations over generations [24, 25]. In 1959 the development of molecular dynamics (MD) simulations [26], which explore atomic motion by integrating Newton's equations, provided a new tool for GO. Here we refer to this kind of method as MD—based global optimization. Simulated annealing (SA), proposed in 1983, introduced a stochastic temperature‐cooling scheme to allow the system to escape local minima [27]. Key advances continued into the 1990s. The first implementation of the Single‐Ended method was reported by Abashkin et al. [28], provided an efficient strategy to locate transition states (TS), facilitating the reaction pathway exploration. Based on this concept, Maeda et al. [29, 30] later developed the global reaction route mapping (GRRM) approach for GO. Particle Swarm Optimization (PSO), in 1995, inspired by collective motion in biological systems, offered a population‐based search strategy [31]. In 1997, basin hopping (BH) transformed the PES into a discrete set of local minima, simplifying the landscape to enable more efficient global exploration [32]. Parallel tempering molecular dynamics (PTMD), in 1999, improved sampling efficiency by allowing structure exchanges between simulations performed at different temperatures [33]. Subsequent years brought a new generation of biologically inspired algorithms. The artificial bee colony algorithm (ABC), introduced in 2005, modeled foraging behavior to optimize structure discovery [34]. Stochastic surface walking (SSW), introduced in 2013, enabled adaptive exploration of the PES through guided stochastic steps that transition between local minima [35]. These innovations, along with the increasing use of machine learning (ML) techniques, have significantly expanded the scope and flexibility of GO strategies. These methods represent the principal strategies used in GO of PES. Over time, they have been progressively refined and adapted to improve efficiency, robustness, and scalability. In recent years, there has been increasing interest in hybrid approaches that combine features from multiple algorithms. As an example, the integration of ML techniques with traditional methods such as GA has demonstrated significant potential to enhance search performance, guide exploration, and accelerate convergence in complex optimization landscapes [36].

Each of these methods offers specific advantages depending on the characteristics of the system under study. Factors such as system size, flexibility, PES complexity, and computational demand all influence the suitability of a given algorithm. Some methods are more effective for small, rigid molecules, while others are better suited to large, flexible, or condensed‐phase systems. Selecting an appropriate GO technique therefore involves balancing accuracy, efficiency, and structural diversity in light of the study's objectives. A defining feature of effective GO is the ability to avoid entrapment in local minima and to continue exploring the PES in search of lower‐energy configurations. To provide a structured framework for the discussion that follows, we classify GO methods into two principal categories: stochastic and deterministic approaches [37, 38]. This classification, here presented for the first time, facilitates a clear comparison of the core strategies and, relative advantages and limitations associated with each algorithm. Stochastic methods incorporate randomness in the generation and evaluation of structures. These algorithms typically begin with random or probabilistically guided perturbations, followed by local optimization to identify nearby minima [10]. The use of non‐deterministic search rules allows these methods to sample the PES broadly and to avoid premature convergence. This makes stochastic algorithms particularly well suited for exploring complex, high‐dimensional energy landscapes. Deterministic methods, in contrast, rely on analytical information such as energy gradients or second derivatives to direct the search toward low‐energy configurations. These approaches follow a defined trajectory based on physical principles and are often capable of precise convergence [39]. However, their reliance on local information and sequential evaluation can make them computationally expensive and less robust in systems with numerous local minima. It is important to note that in the GO literature several authors describe the distinction between deterministic and stochastic methods as follows. Deterministic methods are those that can guarantee the identification of the global minimum with certainty. This process requires exhaustive coverage of the search space, which limits its applicability to relatively small problem instances. In contrast, stochastic methods do not provide such guarantees since they use probabilistic sampling. Although this approach makes them effective for addressing large and complex problems, it does not ensure that a true global minimum will be achieved. Our classification, which emphasizes algorithmic strategies and the role of randomness, aims to underline the potential use of these two approaches in molecular applications.

**FIGURE 3:**
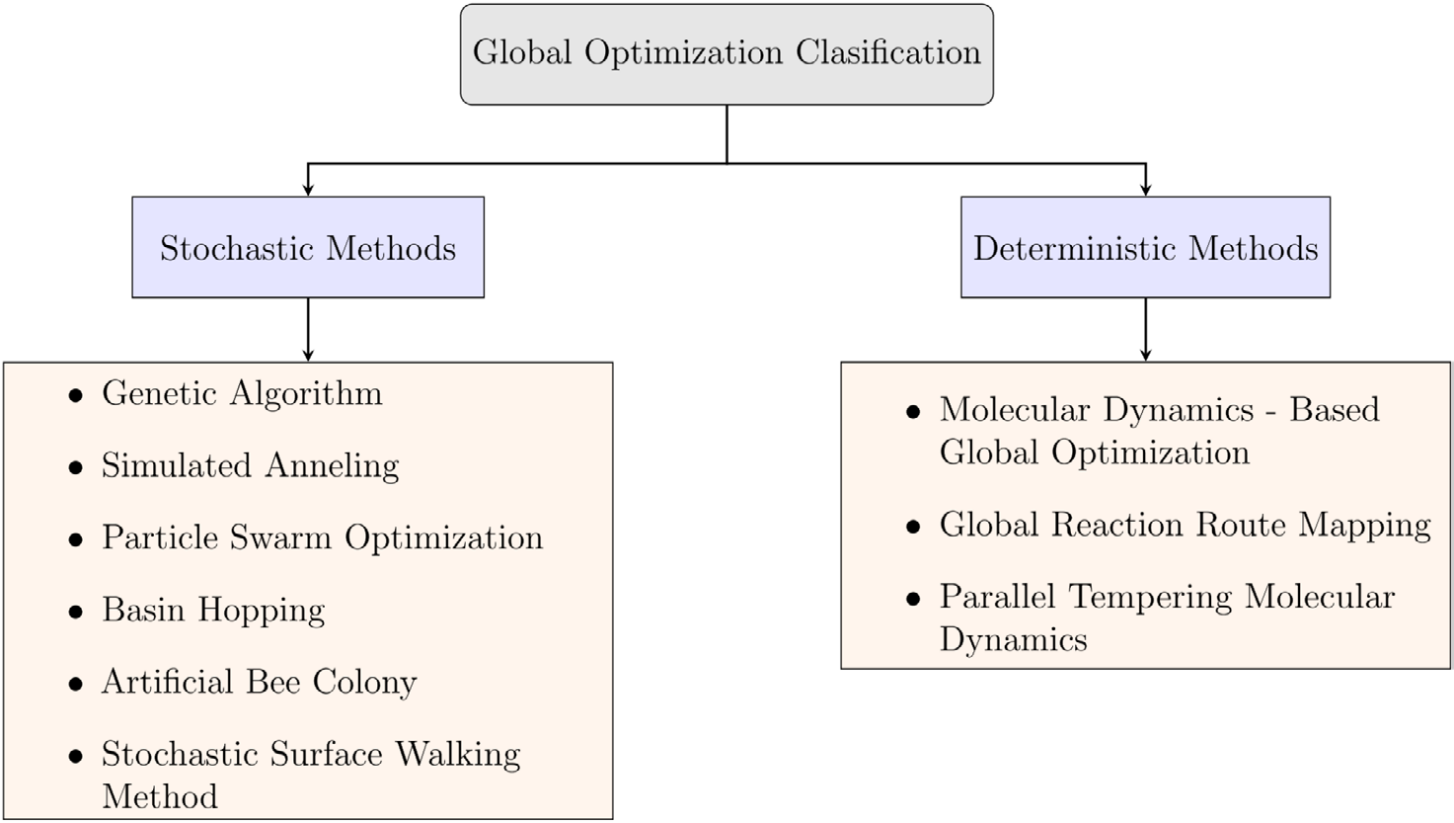
Classification of GO methods into two primary categories: stochastic and deterministic.

As summarized in Figure 3, GO methods can be broadly grouped into these two categories, though hybrid approaches that combine elements of both have also been developed. In the sections that follow, we examine prominent examples within each class. Among stochastic methods, we review GA, SA, PSO, BH, the ABC algorithm, and the SSW method. Among deterministic methods, we discuss MD—Based Global Optimization, the GRRM, and PTMD. Each technique is analyzed in terms of its theoretical basis, algorithmic design, and performance in molecular structure prediction.

### 2.1 Stochastic Methods

Stochastic GO methods identify low‐energy molecular structures by introducing random perturbations to atomic coordinates and applying probabilistic rules to accept or reject new configurations. This process enables the system to move beyond local minima and explore a broader region of the PES [10]. These methods are particularly effective in systems where the PES is high‐dimensional and complex, making exhaustive search impractical.

The theoretical basis for most stochastic algorithms lies in Markov Chain Monte Carlo (MCMC) methods, which combine random sampling with statistically grounded acceptance criteria. A foundational contribution came from Metropolis et al. [40], who proposed a method for sampling configurations according to the Boltzmann distribution at a fixed temperature. Hastings later generalized this approach to allow for more flexible transition probabilities [41]. Central to these algorithms is the Metropolis acceptance criterion, which determines the probability of accepting a move from a configuration with energy $E_{\text{old}}$ to one with energy $E_{\text{new}}$:

(1) $$P_{\text{accept}}=\min\left(1,\exp\left[-\frac{E_{\text{new}}-E_{\text{old}}}{k_{B}T}\right]\right)$$

where T is the effective temperature and $k_{B}$ is Boltzmann's constant. Moves that reduce the energy are always accepted. Those that increase the energy are accepted with a probability that decreases exponentially with the energy difference. This occasional acceptance of uphill moves allows the search to escape local minima. The resulting sequence of configurations forms a Markov chain, where each state depends only on the previous one. Under standard assumptions such as ergodicity and detailed balance, the chain converges to the Boltzmann distribution [42, 43]. Over time, this ensures that sampling becomes independent of the initial configuration and tends to favor low‐energy regions of the PES, while still allowing exploration of higher‐energy states.

To transition from broad exploration to focused refinement, the temperature is gradually reduced according to a predefined cooling schedule [44]. At high temperatures, the algorithm can cross energy barriers and explore distant regions of configuration space. As the temperature decreases, the acceptance of uphill moves becomes less frequent, and the search concentrates within low‐energy basins. When the cooling is sufficiently slow and the number of steps is large, the algorithm converges in probability to the GM [45]. By integrating random sampling, the Metropolis criterion, and temperature control, the stochastic optimization offers a powerful and general strategy for exploring complex PESs. These methods are particularly useful in molecular systems where the configurational space is too large for deterministic or exhaustive methods. In the following subsections, we review several widely used stochastic algorithms in chemistry.

#### 2.1.1 Genetic Algorithm

GAs and related evolutionary computation techniques can be traced back to pioneering computational studies on evolution and adaptation. In 1957, Fraser [24] simulated genetic systems on digital computers, and in the same year Barricelli [25] explored symbiogenetic evolutionary processes through artificial methods. Friedman [46] followed with a digital simulation of evolutionary processes, while Bremermann [47] investigated optimization by means of evolution and recombination. A significant step toward formalizing these ideas came from Holland [48], who proposed a theoretical framework for adaptive systems, laying the conceptual groundwork for computational models of adaptation. In parallel, major advances were taking place in Europe with the development of systematic methods for numerical optimization based on the self‐adaptation of search parameters. Rechenberg [49] and Schwefel [50] pioneered what became known as Evolution Strategies, providing a rigorous basis for evolutionary search methods and influencing subsequent stochastic optimization algorithms. Building on his earlier theoretical research, Holland published his seminal work titled Adaptation in Natural and Artificial Systems in 1975 [51]. This work established the modern GA framework, introducing genetic representations of candidate solutions and variation operators such as selection, crossover, and mutation, inspired by natural evolutionary processes. The first applications of GAs in chemistry emerged in the early 1990s. Tuffery and co‐workers [52] and Lucasius et al. [53] independently developed GA‐based frameworks for molecular amino acids conformational sampling. In 1995, Daeven and Ho [54] use the GA efficiently to find fullerene cluster structures up to C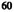 starting from random atomic coordinates. By 1997, Shankland et al. [55] successfully applied to Cartesian‐coordinate GA to determine crystalline pyrene, chlorothiazide and ibuprofen structures starting from powder X‐ray diffraction data. Later, Kowalczyk and collaborators [56] used GAs to predict porosity distributions in amorphous carbon, comparing their performance with numerical and ML approaches. These milestones firmly established GAs as versatile and powerful tools for GO in chemistry and materials science.

**FIGURE 4:**
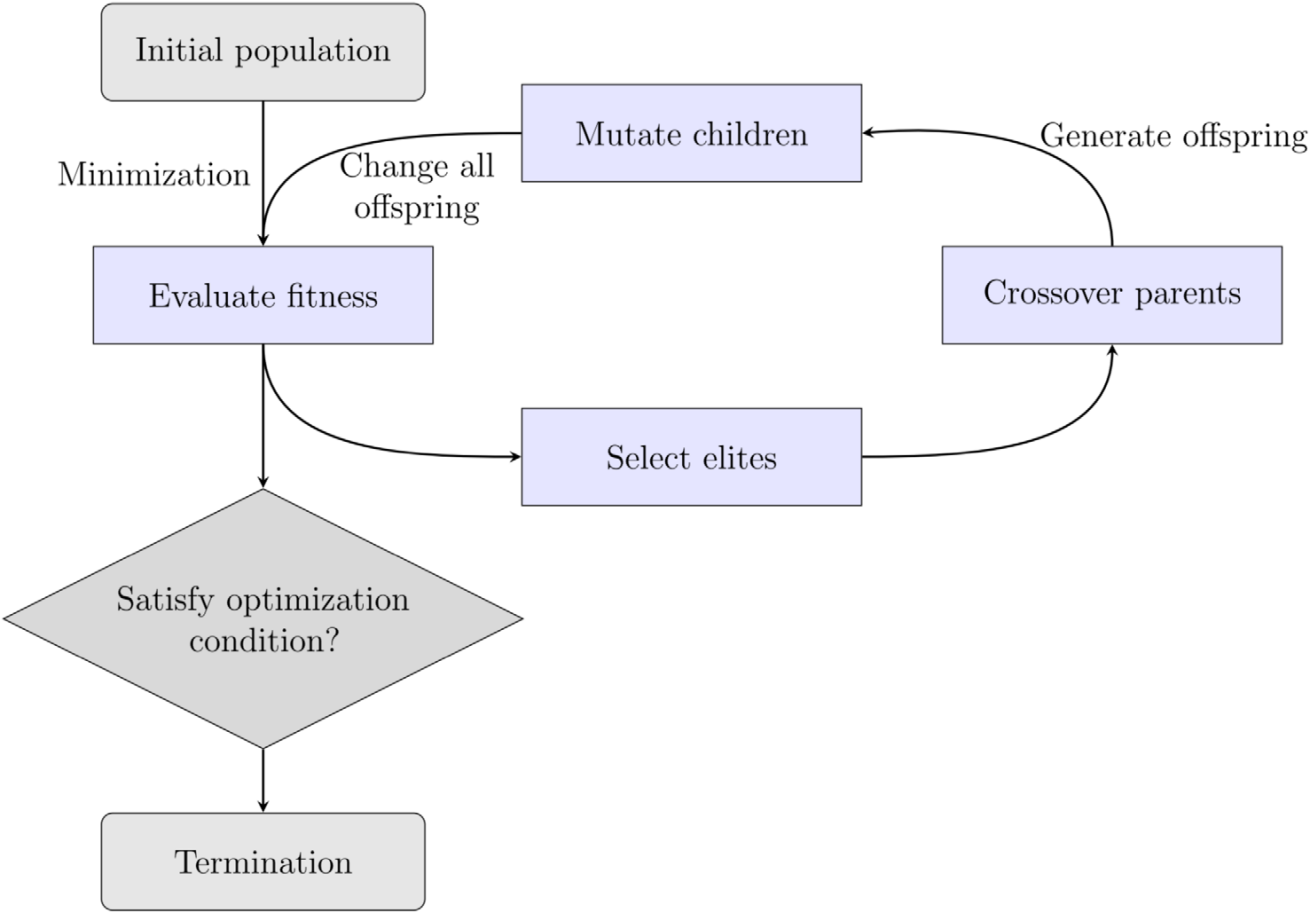
Flowchart illustrating the workflow of a GA applied to GO. See text for details.

GAs simulate the process of natural evolution by applying operations analogous to biological mechanisms, including crossover, mutation, and selection. Figure 4 illustrates a standard GA workflow in the context of molecular or materials optimization. The process begins with a population of candidate structures generated by randomly sampling from a defined chemical space. Each candidate is evaluated using a fitness function, which quantifies its suitability according to a target property, such as potential energy or reactivity. Based on fitness scores, high‐performing individuals are selected to contribute their features to the next generation. Genetic operators are then applied: crossover recombines segments from two parent structures to produce offspring, and mutation introduces random changes to maintain population diversity. The offspring are typically refined using local optimization methods to minimize their energy. After this refinement, a new generation is formed, either by fully replacing the old population or by partially integrating new individuals. This cycle of evaluation, selection, crossover, mutation, and refinement continues until a convergence criterion is met, such as a fixed number of generations or no improvement in fitness over successive iterations. Through this iterative process, GAs are able to traverse complex energy landscapes and identify global minima.

Despite their adaptability, GAs face challenges related to molecular representation. A widely used representation is SMILES [57], which encodes molecular structures as linear strings. Although compact and human‐readable, SMILES strings are highly sensitive to genetic operations. Random crossover and mutation often disrupt the chemical syntax, break ring closures, or violate valency rules, leading to invalid or nonphysical structures [58]. As a result, many generated candidates must be discarded or repaired, reducing search efficiency and increasing computational cost. Furthermore, the non‐local nature of the SMILES encoding means that small syntactic changes can produce large, chemically irrelevant alterations, which further complicates the structure optimization [59, 60]. To address these limitations, alternative molecular representations have been developed. One notable example is SELFIES, a representation designed to be robust to genetic operations [61]. Every SELFIES string corresponds to a valid chemical structure, eliminating the need for extensive validation or repair. Another effective approach involves graph‐based representations, where atoms and bonds are modeled as nodes and edges. This format supports chemically meaningful manipulations and improves interpretability during the optimization process. These alternative encodings significantly enhance the applicability of GAs in molecular and materials discovery. In addition to these symbolic and graph‐based encodings, a landmark contribution by Deaven and Ho [54] demonstrated that genetic operators could be applied directly to three‐dimensional Cartesian coordinates of atomic clusters, entirely bypassing symbolic molecular encodings. This direct‐coordinate approach, although conceptually older, avoids many of the pitfalls of string‐based representations, such as syntax disruption or chemical invalidity, and remains highly effective for structural optimization problems. Even today, Cartesian coordinate GAs are widely used in global structure prediction, often preferred for their simplicity, robustness, and natural compatibility with continuous‐space optimization. In fact, even with the existence of modern encodings such as SMILES, SELFIES, and graph‐based formats, the Cartesian coordinate approach inspired by Deaven and Ho often remains the most straightforward and reliable option for many structural optimization problems [54].

GAs have found widespread use in the design of drug molecules [62], as well as in the GO of atomic clusters [62, 63, 64]. Their parallelizability and compatibility with diverse molecular encodings make them powerful tools for exploring potential energy surfaces. Table 1 highlights ten representative studies that demonstrate the successful application of GAs to GO problems in chemistry and materials science [65, 66, 67, 68, 69, 70, 71, 72, 73, 74].

**TABLE 1:** Set of ten representative applications of genetic algorithm (GA) in molecular systems.

System	GA contribution	GA method	References
AuCu 309‐atom nanoalloy	Determined the most stable stoichiometry and atomic configuration for electrocatalysis	GA with symmetry preserving operators	[65]
IR 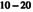 clusters	GO of Ir clusters revealed spin‐dependent global minima and size‐specific cubic motifs	Birmingham Parallel Genetic Algorithm (BPGA) with spin‐polarized DFT calculations	[66]
PdCo nanoalloys	Explored composition‐dependent stability, magnetism, and structure. Also identified Pd segregation and size trends in chemical activity	Birmingham Cluster Genetic Algorithm (BCGA) with DFT and vibrational analysis	[67]
Molecular crystals (blind test targets)	Predicted experimental and new polymorphs	Massively parallel GA with custom operators for crystals and evolutionary niching	[68]
Various small nanoalloy clusters (SNCs)	Reviewed two decades of GA‐DFT development for structural prediction of subnanometer clusters	GA implementations coupled to DFT for free and surface‐supported nanoalloys	[69]
Capsaicinoid analogs (DHCAP and NVA)	Determined solid‐state conformations using GA to identify low‐energy structures	GA with DFT calculations	[70]
Peptide conformers	Efficient sampling of low‐lying peptide conformations and validated against experimental IR spectra	Surrogate‐based Genetic Algorithm (sGADFT)	[71]
Molybdenum‐based catalysts for nitrogen fixation	Discovery of ligands for efficient catalysis	GA with DFT calculations	[72]
Si‐Ge compound semiconductors	Identified low‐energy configurations	GA with DFT and DFPT calculations	[73]
Al 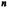 clusters (n = 3‐40)	Explored size‐dependent geometries and binding energies	GA with DFT relaxations on free and supported clusters	[74]

#### 2.1.2 Simulated Annealing

SA is a stochastic GO algorithm inspired by the physical process of annealing in metallurgy, where a material is heated and slowly cooled to allow atoms to rearrange into a low‐energy, defect‐free crystalline structure [27]. This physical analogy provides a conceptual foundation for a computational strategy designed to solve complex optimization problems, particularly those defined by complex energy landscapes containing many local minima [75]. In the SA algorithm, the search begins at a high temperature, allowing both energetically favorable and unfavorable moves to be accepted. This flexibility helps the system escape local minima and explore a broader region of configuration space. As the temperature decreases, the probability of accepting higher‐energy configurations is reduced, gradually focusing the search on low‐energy regions. This controlled transition from global exploration to local refinement makes SA a robust approach for optimization tasks in both discrete and continuous domains.

In molecular structure optimization, SA is commonly applied to minimize the potential energy function E(x), where x denotes the spatial coordinates of the atomic nuclei in an n‐dimensional configuration space. The general procedure of the SA algorithm is outlined in the following six steps given below:

1. **Initialization:** Select an initial molecular configuration x at random direction and assign an initial temperature $T_{0}$. This temperature determines the likelihood of accepting energetically unfavorable moves and sets the scale for configurational exploration.
2. **Perturbation:** Generate a trial configuration $x^{\prime }$ by introducing a random displacement to the current structure. If the k‐th coordinate is selected, its perturbed value is computed as
(2) $$x_{k}^{\prime }=x_{k}+\Delta s\;r_{1}(1-2r)$$
  where r is a uniformly distributed random number in the (0,1) interval, Δs is a user‐defined step size, and the term (1−2r) ensures symmetric sampling of positive and negative displacements.
3. **Evaluation and Acceptance:** Compute the energy of the perturbed configuration, $E^{\prime }=E(x^{\prime })$, and compare it to the energy of the current configuration, E=E(x). If $E^{\prime }<E$, the new configuration is accepted. Otherwise, it may still be accepted based on a probabilistic criterion that depends on the energy difference and current temperature, allowing the system to overcome local barriers.
4. **Update:** If the trial configuration is accepted, it replaces the current one. Otherwise, the current configuration is retained. This process is repeated across the degrees of freedom.
5. **Temperature Reduction:** After a fixed number of iterations or upon reaching equilibrium, reduce the temperature according to a cooling schedule. Common schedules include exponential, linear, or logarithmic decay. This step transitions the algorithm from global search toward local optimization.
6. **Termination:** Continue the process until a stopping condition is satisfied, such as reaching a minimum temperature, exceeding a maximum number of iterations, or observing no significant improvement in energy over a defined number of steps.

**FIGURE 5:**
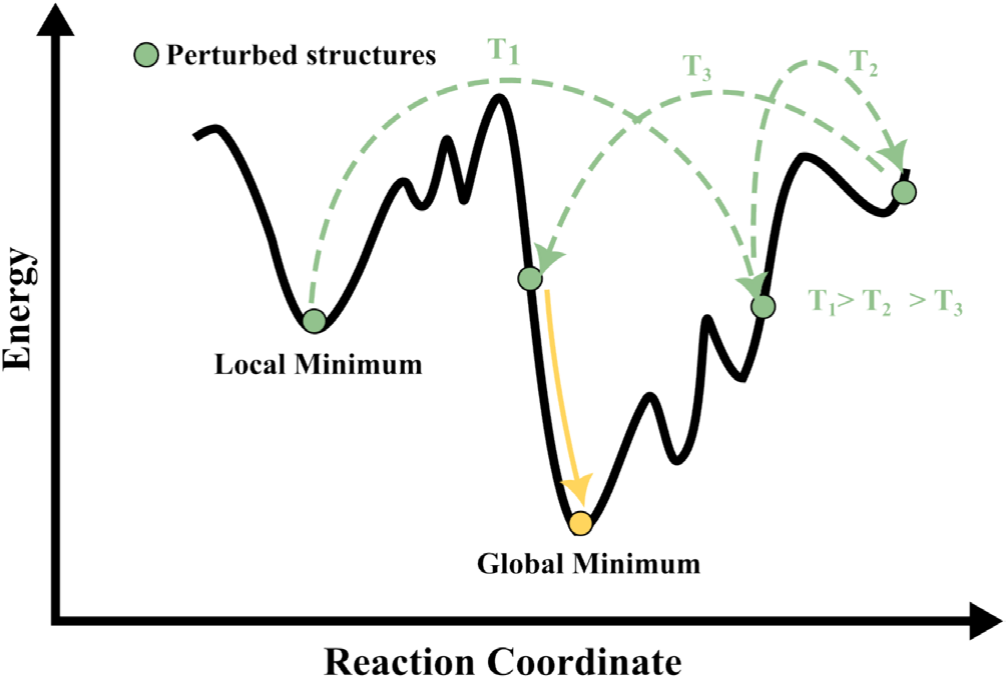
Illustration of the SA algorithm applied to a PES. See text for details.

If the cooling schedule is sufficiently slow and sampling is adequate, SA can identify the GM of the energy landscape. The optimized structure is denoted as $x^{*}$, with energy $E^{*}=E(x^{*})$. A schematic representation is shown in Figure 5, where the black curve represents a complex energy surface with multiple local minima and one GM. At high temperatures ($T_{1}$), the algorithm accepts uphill moves (illustrated by green dashed arrows), facilitating broad exploration. As the temperature decreases ($T_{2}$, $T_{3}$), such moves become less probable, and the search shifts toward local refinement. Green circles mark sampled structures, and the yellow arrow indicates convergence to the GM. This temperature‐controlled balance between exploration and exploitation enables SA to locate the lowest‐energy configuration without becoming trapped in suboptimal regions. SA has been widely applied in molecular geometry optimization, especially for systems with non‐convex energy landscapes that contain many local minima. These cases often involve numerous nearly degenerate configurations, which make GO challenging. SA is effective in sampling such landscapes and identifying low‐energy structures. Table 2 presents selected applications of SA in structural optimization across diverse chemical systems [76, 77, 78, 79, 80, 81, 82, 83, 84, 85].

**TABLE 2:** A set of ten representative applications of simulated annealing (SA) in molecular systems.

System	SA contribution	SA method	References
Water clusters (${\text{H}}_{2}\text{O}$) 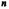 , n=2‐8	Make a GO to demonstrated success for small clusters and structural limitations for larger ones	Diffusion equation‐based deformation of the PES	[76]
GroEL protein complex (E. coli)	Improved structure refinement and conformational analysis	Torsion‐angle dynamics with SA	[77]
46‐residue BLN protein	Located GM	Conformational Space Annealing	[78]
Covalent crystals (boron nitride)	Predicted crystal structures using SA with full ab initio energy evaluations; identified low‐energy candidates	SA	[79]
Peptides and miniproteins	Identifying near native conformations	Multiple Simulated Annealing‐Molecular Dynamics (MSA‐MD)	[80]
Tetrazolate‐based metal‐organic frameworks	Designed and screened 424 MOFs via molecular simulation to identify optimal CH 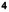 storage candidates	SA	[81]
Lennard‐Jones atomic clusters (25‐40 atoms)	Developed adaptive cooling based on heat capacity	Adaptive SA using MD and heat‐capacity control	[82]
Zeolites	Local minima in periodic systems; improved prediction of interaction energies relevant for adsorption and catalysis	SA with DFT‐based MD in periodic framework	[83]
Tetrazole derivatives on Cu(111) surface	Identified optimal corrosion inhibitors for studied adsorption configurations and electronic descriptors	SA with MD	[84]
Coumarin derivatives on Fe(110) and Cu(111) surfaces	Identified bicoumarin as the optimal inhibitor based on adsorption energy and electronic structure	SA with MD	[85]

In molecular chemistry, SA has been used for conformational analysis of flexible organic molecules [86], and for optimizing molecular clusters composed of organic species [87]. Its ability to overcome energy barriers enables the discovery of low‐energy conformers that are often inaccessible to local optimization techniques. Furthermore, SA has been successfully combined with quantum chemical methods, such as DFT, for the optimization of organometallic systems [88], where the energy landscape is typically multidimensional and highly complex. While SA may not always offer the fastest convergence, its simplicity, robustness, and effectiveness in global search make it a valuable tool in computational chemistry. While SA has a solid theoretical foundation, including a proof that it converges to the GM under an exponential cooling schedule and an infinitely long trajectory [45], this is of limited practical use because the required runtime is prohibitive. Numerous accelerated variants have been proposed to improve efficiency [89, 90], but in large‐scale molecular applications SA is often outperformed by more modern nature‐inspired and hybrid GO strategies. Nevertheless, its conceptual simplicity, robustness, and ability to escape deep local minima have made it a historically important method in molecular structure optimization, and it remains valuable for certain classes of problems, especially in situations where it is necessary to explore a wide range of possible molecular structures.

#### 2.1.3 Particle Swarm Optimization

PSO is a population‐based stochastic algorithm that combines exploration and exploitation strategies to search for global optima in multidimensional spaces. PSO was inspired by the collective behavior observed in biological systems, such as bird flocking and fish schooling [91]. Since its introduction, the algorithm has been adapted [92, 93], modified [94], and parametrized [95], while retaining the core principle of swarm‐based learning and movement toward optimal solutions. In PSO, each particle represents a candidate solution and explores the search space by updating its position and velocity according to both its individual experience (personal best position) and the experience of the entire swarm (global best position). This process enables the swarm to navigate complex potential energy surfaces, effectively avoiding local minima and identifying low‐energy configurations.

The general structure of the PSO algorithm is illustrated in Figure 6. The workflow consists of the following steps:

1. **Initialization:** A swarm of particles is initialized with random positions and velocities within the defined search space.
2. **Fitness Evaluation:** The potential energy of each particle's configuration is computed to evaluate its fitness.
3. **Personal Best Update:** If a particle's current position yields a lower energy than its previous best, the personal best position, $p_{\text{best}}$, is updated.
4. **Global Best Update:** The best position among all particles is identified and stored as the global best position, $g_{\text{best}}$.
5. **Velocity and Position Update:** Each particle updates its velocity and position based on three components:
  - Inertia: the current velocity.
  - Cognitive component: attraction to $p_{\text{best}}$.
  - Social component: attraction to $g_{\text{best}}$.
  **FIGURE 6:**
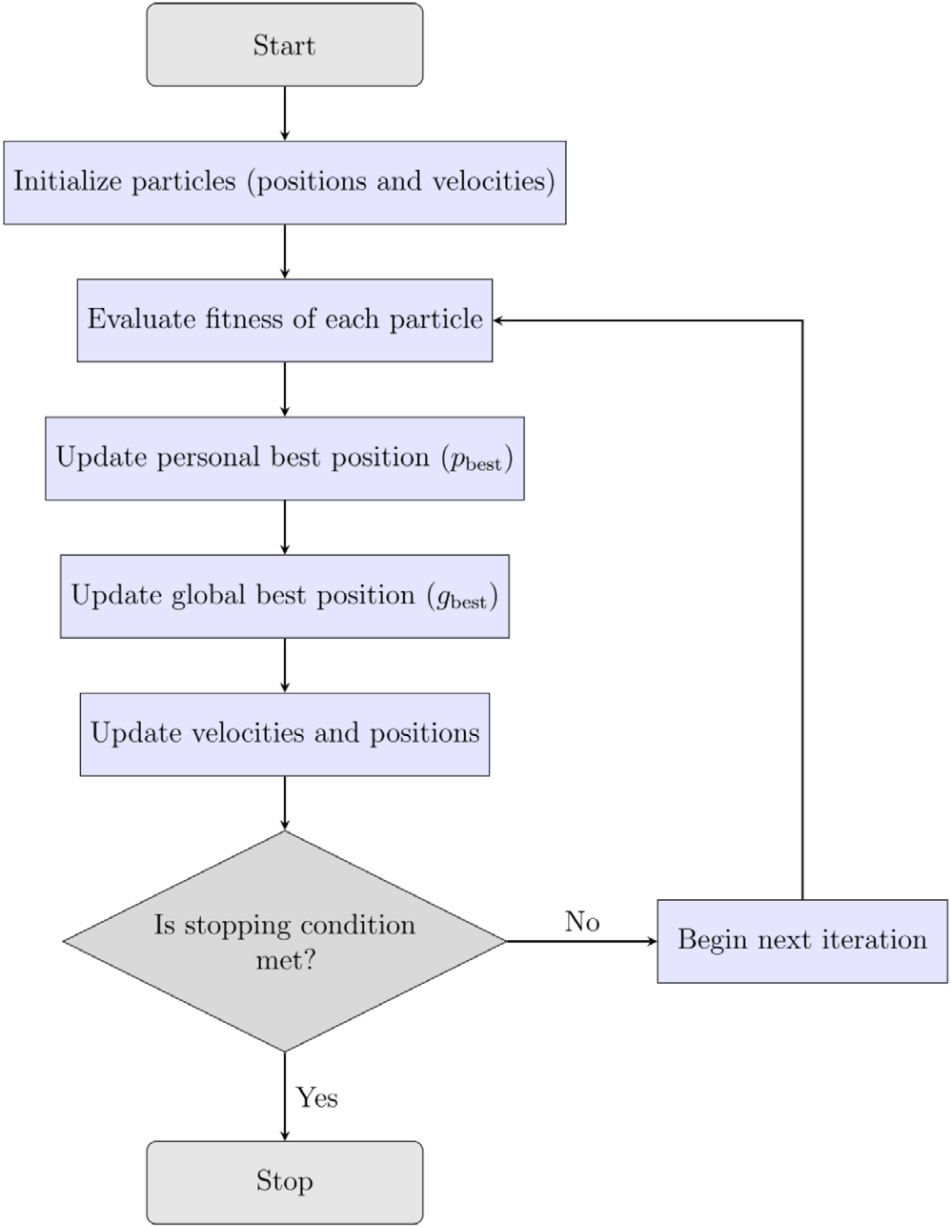
Flowchart representing the PSO algorithm for GO. See text for details.
  These components allow the swarm to balance exploration of new regions with exploitation of known good solutions.
6. **Stopping Condition:** The process repeats until a convergence criterion is met, such as a maximum number of iterations or a minimum energy threshold.

PSO is particularly attractive for problems where gradient information is unavailable, unreliable, or computationally expensive. This includes objective functions that are noisy, non‐differentiable, or highly nonlinear. Its population‐based structure also allows for straightforward parallelization, making it well‐suited for large‐scale molecular optimization. In chemical applications, PSO has been used to study a wide range of systems, including small molecules, atomic clusters, and materials. Table 3 summarizes representative examples, specifying the system type and optimization goals [96, 97, 98, 99, 100, 101, 102, 103, 104, 105]. Due to its simplicity, flexibility, and effectiveness, PSO remains a valuable method for exploring complex potential energy surfaces and identifying global minima in diverse molecular and materials systems.

**TABLE 3:** A set of ten representative applications of particle swarm optimization (PSO) in molecular systems.

System	PSO contribution	PSO method	References
Elemental, binary, and ternary crystals	Global structure prediction	PSO with symmetry constraint and variable‐cell scheme for crystal structure prediction	[96]
Crystals under variable pressure and composition	Developed CALYPSO software for efficient structure prediction using PSO with symmetry, structural filtering, and diversity‐enhancing techniques	CALYPSO: PSO‐based GO with automated crystal structure prediction tools	[97]
Two‐dimensional boron sheets	Structures composed of triangular and hexagonal motifs; relevant for nanotube and fullerene formation	PSO with DFT for 2D material structure prediction	[98]
Cationic water cluster (${\text{H}}_{2}\text{O}$) 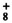	Discovered globally optimized cage‐like structure	PSO with MP2 and DFT	[99]
Drug‐like molecules in lead optimization	Predicted molecular properties in a continuous latent space to design compounds with improved pharmacological profiles	PSO with ML chemical space for multi‐objective molecular design	[100]
Carbon clusters C 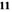 and C 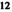	Predicted low‐energy structures	PSO Hartree‐Fock	[101]
Cationic water cluster (${\text{H}}_{2}\text{O}$) 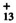	Explored low‐lying structures	PSO with hybrid DFT functionals	[102]
Sub‐nano Ni clusters (gas phase and supported)	Predicted size‐dependent structures and studied oxide support effects and hydrogen‐induced morphology transitions via thermodynamic analysis	PSO with DFT	[103]
1D supramolecular polymer stacks	Generate low‐energy supramolecular stacks with Π‐Π interactions	PSO	[104]
Bzi 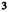 inside carbon nanotubes (host‐guest)	Predict guest orientation in Bzi 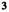 @(m,m)	PSO‐LJ method as pre‐screening for DFT host‐guest orientation prediction	[105]

#### 2.1.4 Basin Hopping

**FIGURE 7:**
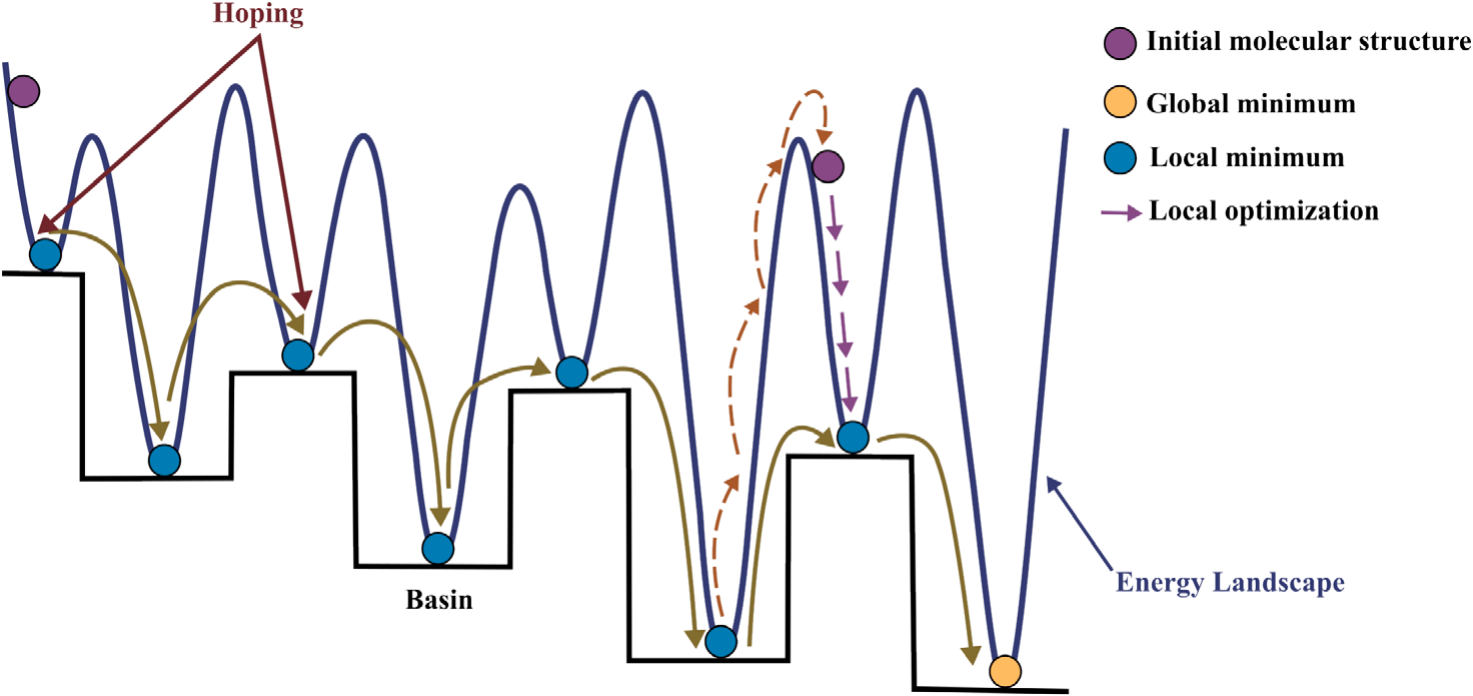
Illustration of the BH algorithm applied to a complex PES. See text for details.

BH was applied it to the structural optimization of Lennard‐Jones clusters and demonstrated its effectiveness in identifying low‐energy configurations on complex PES [32]. The central idea of BH is to transform the continuous PES into a landscape composed of local minima, or “basins”, by associating each molecular configuration with its nearest minimum via local geometry optimization. It has to be noticed that this strategy is not exclusive to BH, as it is also used by other GO algorithms. This conceptual transformation simplifies the global search by filtering out high‐frequency vibrational modes and focusing on basin connectivity rather than the full curvature of the PES. Figure 7 provides a schematic representation of the BH algorithm applied to a multidimensional PES. The blue curve illustrates the original landscape, which contains numerous local minima separated by energy barriers. At each iteration, the system is randomly perturbed (purple points), and a local minimization is performed to locate the nearest basin (blue points). This maps the PES onto a transformed, stepwise landscape where only the energies of minimized configurations are considered. The search proceeds by “hopping” between basins (solid arrows), and when trapped in a deep local minimum, additional random displacements (dashed arrows) enable the system to escape. The combination of stochastic perturbation, deterministic local minimization, and a Metropolis‐type acceptance criterion based on minimized energies [40] enables BH to efficiently explore the configurational landscape and identify the global minimum (orange point).

The conceptual basis of BH aligns with earlier methods that operated in the discrete space of minima, such as eigenvector‐following techniques used to construct pathways between stationary points [106]. A related idea was proposed by Barkema and Mousseau [107], who explored basins of attraction in disordered systems. While these approaches provided valuable insight into configurational connectivity and reaction mechanisms, their reliance on repeated transition state searches limited their scalability. BH, by contrast, bypasses explicit transition state evaluation and instead operates directly in configuration space. It stochastically samples basins through perturbation and accepts or rejects new configurations based on the energy of the minimized structure. This approach allows efficient global sampling while maintaining a focus on local minima, offering a practical balance between computational efficiency and physical relevance. Although BH does not explicitly recover transition state information, it captures the essential features of the energy landscape that govern thermodynamic stability. The method can be coupled with a wide range of energy models, from empirical force fields to DFT. BH has been successfully applied to atomic and molecular clusters [108], biomolecular folding, supramolecular assemblies, and surface‐adsorbate systems. It is particularly effective for systems characterized by complex energy landscapes with numerous local minima, where traditional optimization methods often become trapped. Thousands of applications have been reported, and several extensions of the basic algorithm have been proposed [109, 110].

Over the years, various enhancements have improved the performance and applicability of BH. These include biasing strategies to prevent revisiting known basins, adaptive step‐size tuning, and integration with ML potentials to accelerate local minimization [111]. In more recent studies, BH has also been embedded into multi‐level search protocols and coupled with other GO methods, such as GA or metadynamics, to further improve configurational sampling. Due to its robustness, versatility, and ease of implementation, BH remains a standard method for GO in computational chemistry. Table 4 summarizes ten representative applications in which BH has contributed to the identification of global or near‐global minima across a range of chemical systems, including atomic clusters, host‐guest complexes, and crystalline frameworks [112, 113, 114, 115, 116, 117, 118, 119, 120, 121].

**TABLE 4:** Set of ten representative applications of basin hopping (BH) in molecular systems.

System	BH contribution	BH method	References
Si 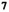 , Cu 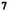 , Si 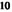 clusters	Identify all low‐energy isomers within 1 eV of the GM	Compared two types of atomic displacements (single‐atom and all‐atom)	[112]
Water, methanol, and protonated water/methanol clusters	Identify global minima without relying on empirical force fields	BH implemented with DFT, dispersion found critical for methanol conformers	[113]
Tungsten clusters W 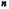 (n=2‐16, 30‐120)	Determine stable geometries and electronic properties of small and large W clusters	Combined BH with tight‐binding and Finnis‐Sinclair potentials; validated structures with DFT	[114]
Hydroxylated silica nanoclusters (${\text{SiO}}_{2}$) 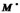 (${\text{H}}_{2}\text{O}$) 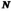 (M=6‐12)	Identify global minima and hydroxylation trends	Cascade Monte Carlo BH using two interatomic potentials and final DFT refinement	[115]
Metal and boron clusters (gas phase and surface‐supported)	Identify global minima of isolated and supported clusters using efficient search strategy	TGMin software with constrained BH: includes displacement rules, planarity filters, ultrafast shape recognition, and morphology‐based selection for divide‐and‐conquer optimization	[116]
Polymorphic organic molecular crystals	Estimate energy barriers and landscape connectivity between polymorphs	Monte Carlo threshold BH algorithm applied to crystal landscapes	[117]
CuP 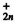 cluster cations (n=2‐11)	Identify global minima and predict dissociation channels	BH with custom NKCS code + DFT	[118]
Crystalline benzene polymorphs	Predict all known polymorphs using an anisotropic potential model	BH with variable cell size and molecular orientation; unbiased crystal structure prediction using Ewald summation and supercell updates	[119]
Pt single‐atom catalyst	Determine active‐site geometry under varying ligand and electrochemical potential conditions	Grand canonical BH with DFT	[120]
${\text{Ba}}_{0.5}$ ${\text{Sr}}_{0.5}$ ${\text{Co}}_{0.8}$ ${\text{Fe}}_{0.2}$ ${\text{O}}_{3}$, ${\text{Cs}}_{0.2}$ ${\text{Sr}}_{0.8}$ ${\text{Co}}_{0.4}$ ${\text{Fe}}_{0.6}$ ${\text{O}}_{3}$	Optimization of cation ordering in complex perovskites with on‐the‐fly trained ML potentials	Recommender‐based BH (RBH) with Gaussian Approximation Potentials (GAP) trained during BH iterations	[121]

#### 2.1.5 Artificial Bee Colony

ABC algorithm is a population‐based metaheuristic inspired by the foraging behavior of honeybee colonies. The method has since been applied to a wide range of optimization problems [34], including those in engineering design, ML, and bioinformatics [122, 123]. In the ABC framework, each food source represents a candidate solution in the search space, and the nectar quantity corresponds to its objective function value or fitness. The algorithm seeks to identify the most productive food source, which represents the global optimum. The colony comprises three types of artificial agents: employed bees, onlooker bees, and scout bees. Each employed bee is associated with a specific food source and conducts a local search in its neighborhood, updating the food source if a better solution is discovered. Fitness information is shared with the hive, where onlooker bees probabilistically select food sources based on their quality and perform additional local searches. If a food source fails to improve over a predefined number of iterations, it is considered exhausted. The corresponding employed bee becomes a scout bee, which explores the search space randomly to introduce new candidate solutions and maintain diversity within the population. This division of roles allows the ABC algorithm to balance global exploration (via scouts) and local exploitation (via employed and onlooker bees), helping prevent premature convergence and supporting thorough search of the landscape [124, 125].

**FIGURE 8:**
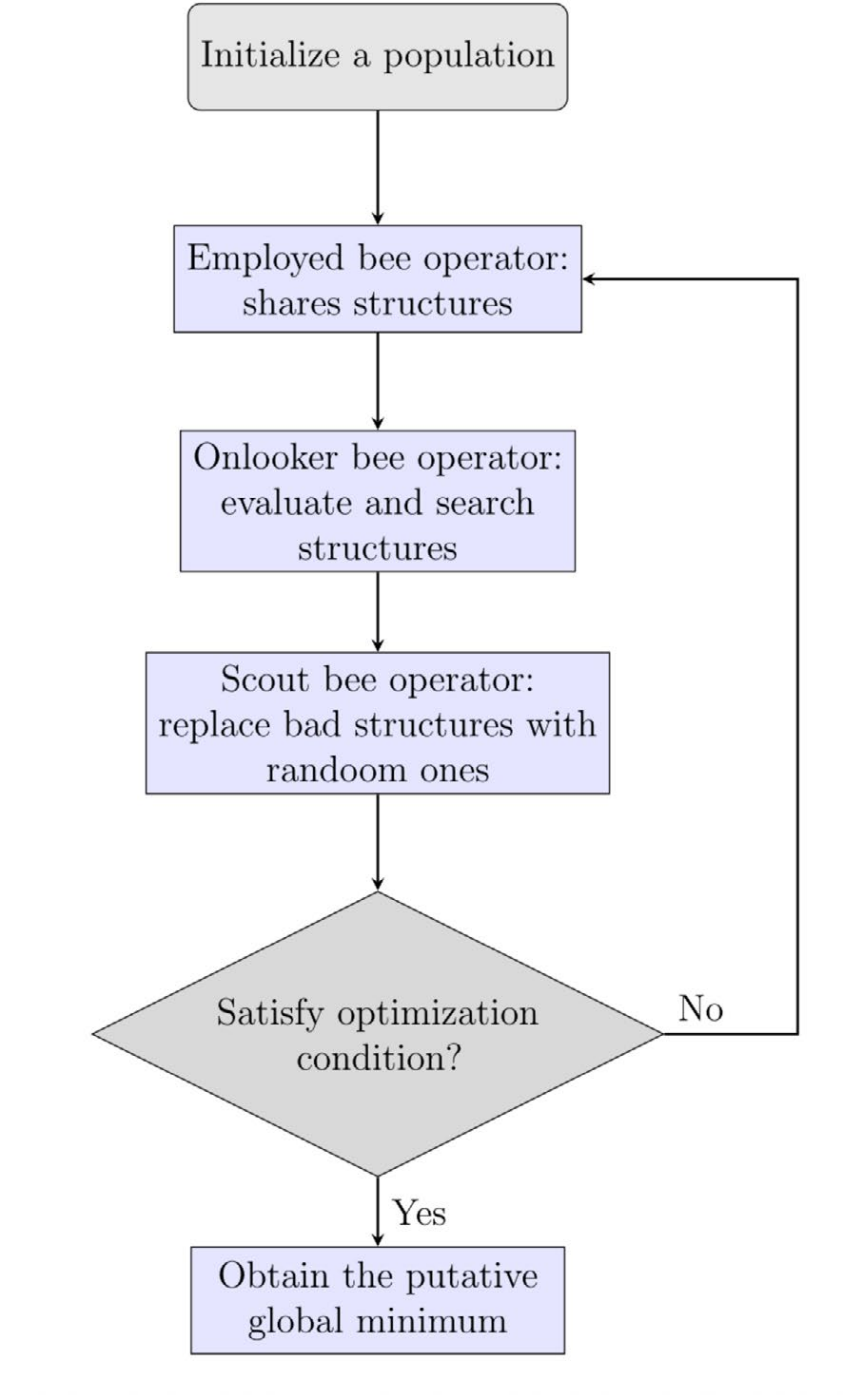
Flowchart of the ABC algorithm for GO. See text for details.

Figure 8 illustrates the operational cycle of the ABC algorithm. The process begins with the initialization of a random population. Employed bees generate new solutions around their assigned food sources and share fitness values with the onlooker bees. Onlookers select promising sources with a probability proportional to their fitness and refine them further. When a food source stagnates, scout bees introduce new randomly generated candidates to refresh the population. This cycle continues until a predefined stopping criterion is met, such as convergence or a maximum number of iterations, after which the best solution found is reported. The ABC algorithm is valued for its simplicity, ease of implementation, and limited number of control parameters. Unlike many other swarm‐based methods, it requires only a few hyperparameters (typically the colony size and the trial limit), yet achieves competitive performance across a broad spectrum of optimization tasks. Its flexible design has also led to the development of numerous variants and hybridizations tailored to specific problem classes. Table 5 summarizes representative examples, specifying the system type and optimization goals [126, 127, 128, 129, 130, 131, 132, 133, 134, 135].

**TABLE 5:** Set of ten representative applications of artificial bee colony (ABC) method in molecular systems.

System	ABC contribution	ABC method	References
Cu 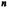 Ni 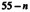 nanoparticles (n = 0‐55)	Explored stability, core‐shell preferences, and magnetic/electronic properties using	ABC algorithm with DFT optimization	[126]
Pt 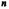 clusters (n = 2‐150)	Identified structural trends and non‐icosahedral motifs consistent with DFT re‐optimization	Modified ABC with DFT calibrated empirical potentials	[127]
Au 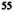 , Au 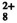 , Au 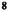 on oxide, [CO 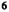 Te 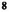 (PEt 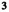 ) 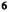 ] [C 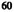 ] 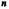 clusters	Introduced an adaptive‐learning ABC algorithm	Adaptive ABC algorithm	[128]
(VH 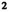 ) 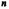 nanoclusters (n = 10‐30)	Identified Kubas interactions and stability trends in specific cluster sizes	ABC algorithm with DFT optimization and bonding analysis	[129]
(Y 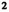 O 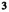 ) 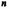 clusters (n = 1‐15)	Identified new medium‐to‐large yttrium oxide nanocluster structures	ABC algorithm combined with DFT calculations and thermodynamic property evaluation	[130]
YGe 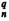 clusters (n = 4‐20, q=0,−1)	Explored size‐dependent evolution of structural, electronic, and spectral properties	ABC method combined with DFT and photoelectron spectra simulation	[131]
Pt 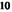 , Sn 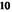 , and Pt 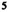 Sn 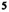 clusters	Investigated ethanol adsorption and OH dehydrogenation reactivity; identified Pt 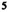 Sn 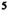 as catalytically superior	ABC algorithm with DFT and QTAIM analysis	[132]
MSn 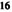 (M = Sc, Y, La) clusters	Identified Frank‐Kasper cage structures	ABC algorithm combined with DFT	[133]
LaGe 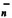 (*n* = 3‐14) clusters	Identified LaGe 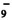 as a magic number cluster with high stability	ABC algorithm with DFT	[134]
Organic molecules (gas and crystal phases)	Predicted diverse conformers addressed conformational mobility and crystal packing challenges	ABC algorithm with RMSD‐based fitness in periodic and non‐periodic systems	[135]

In summary, the ABC algorithm offers a robust, adaptive, and computationally efficient approach to GO. By mimicking the decentralized intelligence of natural foraging behavior, it effectively balances intensification and diversification, making it a widely adopted tool in the field of nature‐inspired computing.

#### 2.1.6 Stochastic Surface Walking

SSW method was used upon their earlier work on continuous transition state (TS) searches using the biased potential‐driven constrained Broyden dimer method (BP‐CBD) [136, 137]. SSW combines bias‐potential‐driven dynamics [138] with the Metropolis Monte Carlo algorithm [40] to facilitate the smooth evolution of a system from one local minimum to another on the PES. At each step, structural perturbations are proposed and then accepted or rejected based on their energetic favorability, enabling efficient exploration of complex energy landscapes. SSW is particularly effective for systems characterized by high‐dimensional, disordered, or structurally flexible PESs, including nanoclusters, amorphous solids, and catalytic interfaces [35]. Each Monte Carlo step in the algorithm consists of three main phases: (1) a climbing phase in which the system is displaced along a low‐curvature direction, (2) a local minimization on a bias‐modified PES, and (3) an acceptance or rejection step based on the Metropolis criterion. The climbing direction is constructed as a hybrid of a global random vector and a localized structural perturbation, refined through the biased dimer rotation technique. During this climb, Gaussian functions are iteratively added to the PES, reducing barriers and facilitating transitions between local basins.

**FIGURE 9:**
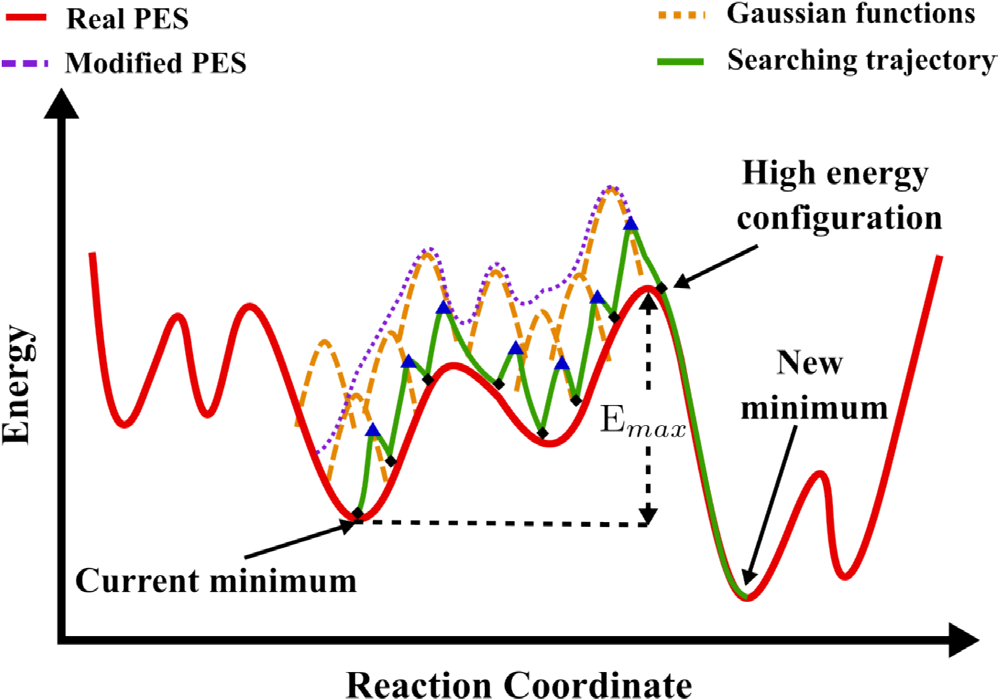
Schematic representation of the SSW method used for global PES exploration. See text for details.

Figure 9 illustrates the conceptual framework of the SSW method. The red curve represents the original PES, while the orange dashed lines show the sequential Gaussian bias potentials. These accumulate to produce a modified PES, shown as the purple dotted line. The green trajectory traces the system as it escapes from an initial minimum, ascends over a barrier, and relaxes into a new minimum. The highest energy reached, $E_{\max}$, serves as an estimate of the transition barrier between the two minima.

The algorithm proceeds through the following steps:

1. **Initialization:** Start from a local minimum $R_{i}^{m}$ and estimate a soft vibrational mode using either dimer rotation or an approximate Hessian.
2. **Mode Selection:**
(3) $$N_{i}^{0}=\frac{\lambda \;N_{i}^{g}+|\lambda |\;N_{i}^{l}}{\left\|\lambda \;N_{i}^{g}+|\lambda |\;N_{i}^{l}\right\|}$$
  where λ∈[0.1,1.5] is randomly selected, $N_{i}^{g}$ is a global mode sampled from a thermal distribution (e.g., 300 K), and $N_{i}^{l}$ is a local mode designed to describe potential bond rearrangements. The direction is then refined via biased dimer rotation.
3. **Biased Climb (for** n=1 **to** H **steps):**
  a Add a Gaussian bias potential:
(4) $$v_{n}(R)=w\exp\left[-\frac{{\left((R-R_{t}^{n-1})\cdot N_{i}^{n}\right)}^{2}}{2ds^{2}}\right]$$
    where w and ds are the Gaussian height and width, respectively.
  b Modify the PES:
(5) $$V_{\text{mod}}(R)=V_{\text{real}}(R)+\overset{n}{\underset{k=1}{\sum }}v_{k}(R)$$
  c Displace the system along $N_{i}^{n}$ by a step ds, then locally minimize on $V_{\text{mod}}$ to obtain the updated structure $R_{t}^{n}$.
  d Refine the search direction $N_{i}^{n+1}$ using dimer rotation at $R_{t}^{n}$.
4. **Unbiased Relaxation:** Remove all Gaussian bias terms and minimize the final configuration $R_{i}^{H}$ on the original PES $V_{\text{real}}$.
5. **Metropolis Acceptance:** Accept or reject the new minimum using the Metropolis criterion based on its energy relative to the previous structure.
6. **Iteration:** Repeat the full procedure to ensure thorough sampling of the PES.

Although SSW does not explicitly identify transition states, it shares conceptual similarities with eigenvector‐following techniques [32] by implicitly mapping out transition pathways between minima. This makes it particularly valuable for systems where barrier crossing is essential. The method has demonstrated efficiency in exploring PES topologies and locating low‐energy structures for covalent molecules, Lennard‐Jones clusters [139], and Morse‐type systems. SSW has since been applied to a variety of chemical and materials systems, including the prediction of nanocluster geometries, amorphous carbon phases, and solid‐state phase transitions. Table 6 summarizes ten representative studies employing SSW for such tasks. Importantly, SSW not only identifies thermodynamically stable structures but also preserves the intermediate trajectory, which can be analyzed post hoc to extract transition states and construct kinetic networks. Recent developments include the integration of neural network potentials (SSW‐NN) [140], which significantly accelerate energy evaluations while maintaining near ab initio accuracy. This hybrid approach extends the applicability of SSW to larger and more complex systems without compromising physical fidelity. Table 6 presents ten representative studies where SSW has been applied to global minimum searches [141, 142, 143, 144, 145, 146, 147, 148, 149, 150].

**TABLE 6:** Set of ten representative applications of stochastic surface walking (SSW) method in molecular systems.

System	SSW contribution	SSW method	References
TiO 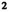 phase transition	Identified TiO 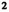 ‐II thin slab as the kinetically preferred nucleation phase on anatase (112) surface; revealed role of strain and directionality on transition barriers	SSW‐based pathway sampling	[141]
ZrO 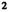 tetragonal‐to‐monoclinic phase transition	Resolved atomistic mechanism and identified two near‐degenerate transition pathways and a stress‐induced ferroelastic channel	SSW‐based PES exploration and transition pathway sampling using first‐principles methods	[142]
Pt_*N*_ subnano clusters (N = 12‐46)	Identified 20 new global minima and revealed structural evolution from amorphous to core‐shell architectures	SSW global search using parallel PES exploration for DFT optimized structures	[143]
TiO 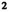 crystal polymorphs	Discovered two new porous TiO 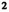 structures with comparable stability to rutile	SSW‐NN method combining DFT generated global dataset with atom‐centered neural networks	[144]
Water‐gas shift on Cu(111)	Identified water dissociation as rate‐determining and formic acid as a key intermediate	SSW‐Cat method combining SSW‐based global conformer search	[145]
Lithiation of anode materials	Discovered metastable 3D tunnel TiO 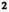 (TiO 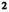 ‐S) phase with low volume expansion and fast Li diffusion	SSW‐based high‐throughput structural evolution sampling for lithiation	[146]
Yttria‐stabilized zirconia	Developed global NN potential for Y‐Zr‐O system	SSW‐NN GO with trained Y‐Zr‐O G‐NN potential	[147]
Hydrocarbons on Cu(111) surface	Developed Cu‐C‐H neural network potential to explore stable hydrocarbons and dissociation pathways; identified ring stabilization by hydrogen	SSW‐NN with deep potential training and free energy surface analysis via metadynamics	[148]
Cu 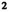 O/Cu interface and CO 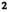 reduction	Identified metastable hcp‐Cu formation on Cu 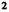 O surface with superior CO 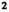 reduction performance using SSW‐NN phase exploration	SSW‐NN global structure search under oxygen vacancy‐driven reduction conditions	[149]
Borophene on Ag(100) substrate	Mapped nanometer‐scale structural diversity and polymorphism via large‐scale SSW search integrated with ML potentials	SSW GO with neural network potentials and AL on 556 supercells	[150]

In summary, the SSW method offers a robust, unbiased, and computationally efficient framework for global exploration of complex PESs. Its ability to simultaneously provide both structural and kinetic insights makes it a powerful tool for understanding phase transitions, chemical reactivity, and structural evolution in a wide range of molecular and materials systems. It is important here to underline that the choice of PSO, ABC, and SSW is based on the fact that they represent nature‐inspired paradigms that rank among the most common and successful approaches for the GO of molecular structures [31, 34, 35]. Their widespread adoption reflects their proven ability to navigate high‐dimensional PES, balance exploration and exploitation, and consistently achieve strong performance in locating low‐energy structures and exploring complex PES landscapes in chemical applications.

### 2.2 Deterministic Methods

Deterministic GO methods provide a mathematically rigorous framework for locating the global minimum of a PES within a specified numerical tolerance [151]. Unlike stochastic algorithms, which rely on random sampling and probabilistic decision‐making, deterministic methods guarantee convergence by systematically partitioning and bounding the search space [152]. This level of determinism is particularly valuable in computational chemistry, where the accurate identification of molecular structures, reaction intermediates, or cluster geometries often depends on subtle energetic differences and demands reproducibility and verifiable precision. Deterministic approaches have been most successful in small to medium‐sized systems, where the number of degrees of freedom remains computationally tractable. Applications include conformational searches of flexible molecules [39], rigorous determination of global minima in atomic clusters, and the calibration of potential energy surfaces for reactive chemical systems. These methods are especially well‐suited for benchmarking empirical force fields or validating quantum chemical predictions, where energy differences between candidate structures may be on the order of a few kcal/mol, and definitive identification of the thermodynamic ground state is critical.

A principal challenge associated with deterministic methods lies in their unfavorable scaling with system dimensionality. The computational cost increases exponentially with the number of degrees of freedom, restricting practical applications to lower‐dimensional systems unless this cost is mitigated by techniques such as coordinate transformation, symmetry exploitation, or the incorporation of surrogate models [153, 154]. Despite this limitation, the ability of deterministic methods to yield certified global optima renders them indispensable in scenarios where accuracy, interpretability, and reproducibility are paramount. These include drug conformer enumeration, mechanistic pathway discovery, and global PES mapping in spectroscopic applications. Recent advances in interval analysis, adaptive spatial decomposition, and domain‐specific bounding strategies have significantly expanded the scope of deterministic global methods. As computational resources continue to grow and algorithmic efficiencies improve, deterministic methods are expected to play an increasingly important role in high‐accuracy molecular structure prediction and global reactivity analysis. In this section, we review several prominent deterministic approaches and highlight their application to chemically relevant systems, focusing on their strengths, limitations, and integration with other optimization paradigms.

#### 2.2.1 Molecular Dynamics—Based Global Optimization

MD trajectories can be used as tool for a GO protocol. In this context when configurations from the trajectory are systematically selected and subsequently locally optimized to nearest minima. This combined approach enables the identification of low‐energy structures across different regions of the PES. The simulation begins with a defined set of atomic positions and velocities, from which the subsequent trajectory is fully determined [155]. While an exact treatment would require solving the coupled quantum dynamics of both nuclei and electrons, practical implementations typically employ the Born‐Oppenheimer approximation [156]. Under this approximation, referred to as Born‐Oppenheimer molecular dynamics (BOMD), the electronic structure is computed for fixed nuclear coordinates at each time step, and the resulting forces are used to propagate the nuclei according to Newtonian mechanics. BOMD provides an effective framework for exploring PES, particularly when chemical accuracy is required to capture bond rearrangements and electronic structure variations. This approach differs from classical MD, which relies on empirical force fields, and from Car‐Parrinello molecular dynamics (CPMD), which treats both electrons and nuclei as dynamical variables coupled through a fictitious mass parameter [157]. Among these methods, BOMD offers a balance between computational efficiency and quantum‐level accuracy, making it a valuable tool for structure prediction and energy landscape exploration in finite molecular systems.

**FIGURE 10:**
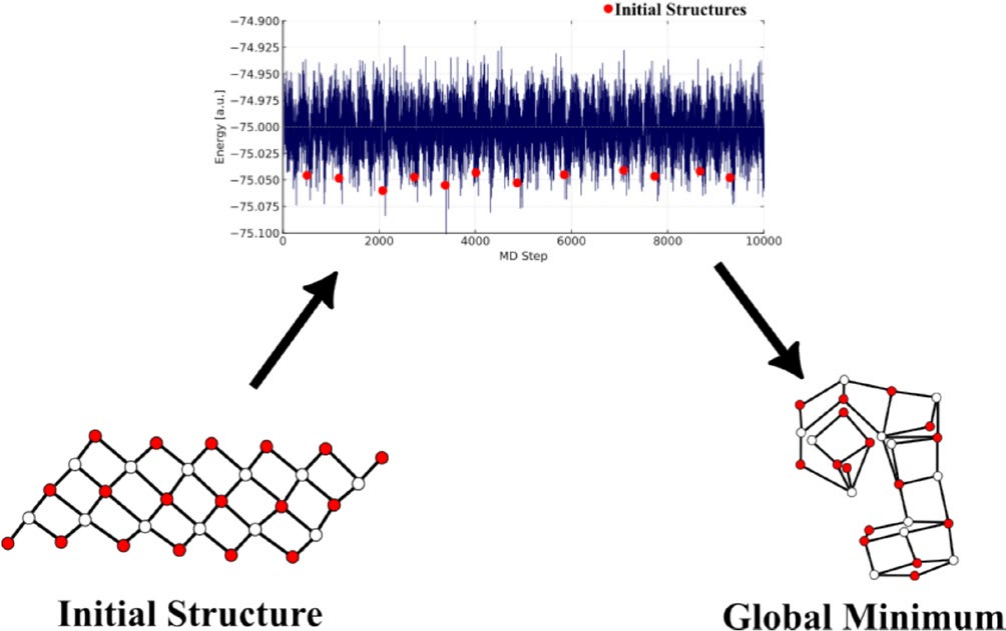
Schematic representation of molecular dynamics—based global optimization procedure. See text for details.

A typical GO workflow using BOMD begins from a known local minimum. Initial atomic velocities are sampled from a Boltzmann distribution at an elevated temperature, which must be high enough to promote transitions between minima, yet low enough to avoid unphysical bond breaking or fragmentation. Thermostats such as the Langevin [158] or Nosé‐Hoover [159] algorithms are commonly employed to maintain the target temperature and ensure sampling of the canonical ensemble. In cases where pressure control is needed, barostats may also be applied. Once initial conditions are set, the system is propagated forward in time according to Newton's equations of motion. At each time step, interatomic forces are computed via quantum mechanical methods, yielding a trajectory that samples different regions of the PES. Figure 10illustrates a schematical representation of this process. Low‐energy molecular configurations sampled from the MD trajectory are selected. These structures are then locally optimized to refine them to their nearest energy minima. By comparing the energies of these locally optimized structures, one can identify the GM or other low‐energy candidates. One common limitation in BOMD‐based exploration is the relatively slow movement of heavier atoms, which can impede efficient sampling of the PES. To address this, the scaled‐mass BOMD (or scale‐BOMD) technique reduces the masses of heavier elements, thereby accelerating their dynamics without significantly altering the equilibrium structure [160, 161, 162]. Although this approach modifies the momentum distribution, it often leads to more effective exploration within a fixed simulation time.

Beyond conventional MD simulations, enhanced sampling methods, such as metadynamics, have been widely employed to accelerate the exploration of the PES by facilitating the crossing of high free‐energy barriers [163, 164, 165, 166, 167, 168, 169, 170, 171, 172]. In metadynamics, a history‐dependent bias potential is iteratively added to selected collective variables, discouraging the system from revisiting previously explored configurations and thereby enabling efficient sampling of rare events [163, 164, 165]. This technique has been successfully applied in diverse contexts relevant to MD‐based global optimization, including the exploration of complex reaction pathways, such as those in Lennard‐Jones and water clusters [166], the prediction of polymorphic crystal structures, such as zeolites [167, 168], and the refinement of biomolecular conformations [169]. For example, metadynamics has been used to identify transition states in catalytic systems [170], to explore nucleation mechanisms in crystallization, and to map free‐energy surfaces in protein‐ligand binding studies [171]. These approaches, along with related enhanced sampling techniques such as replica‐exchange metadynamics [172] and bias‐exchange metadynamics, represent powerful tools for improving the efficiency and robustness of MD‐based global optimization strategies.

BOMD has been successfully employed in a wide range of chemical applications, including structure prediction of Bi
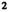
O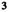 clusters [160], nickel‐palladium bimetallic clusters [173], sodium heptamer clusters [174], and copper‐palladium nanoalloys [175]. These studies highlight the utility of BOMD in accessing reactive and flexible regions of the PES with quantum mechanical accuracy. Table 7 summarizes additional examples of MD‐based global optimization protocols in chemically relevant systems [160, 161, 176, 177, 178, 179, 180, 181, 182, 183]. These include pure BOMD and hybrid approaches that integrate MD with other GO techniques, such as BH, Monte Carlo sampling, and GA. As computational power continues to increase, MD‐based strategies are expected to play an expanding role in molecular structure prediction, reaction pathway discovery, and the generation of thermodynamically relevant structural ensembles.

**TABLE 7:** Set of ten representative applications of molecular dynamics based global optimization method in molecular systems.

System	MD contribution	MD method	References
(Bi 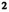 O 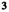 ) 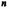 clusters (n = 1‐5)	Explored ground‐state and low‐lying isomer structures	Scaled‐BOMD with ADFT	[160]
(Bi 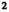 O 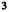 ) 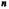 clusters (n = 6‐9)	Identified ground‐state and low‐lying isomers using BOMD‐generated structures as input for local optimizations	BOMD with ADFT	[176]
(Fe 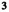 O 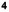 ) 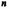 clusters (n = 1‐5)	Determined ground‐state structures of neutral, cationic, and anionic species	Scaled‐BOMD with DFT	[177]
MPd 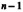 clusters (M = Ni, Cu; n = 2‐13)	Explore ground‐state and isoenergetic structures of doped palladium clusters, revealing Ni doping enhances stability and reactivity	BOMD with ADFT	[161]
O 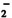 (H 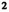 O) 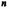 clusters (n = 1‐16)	Explore charge distribution and stability in hydrated anionic clusters; identified magic‐number clusters with compact hydrogen‐bonded networks	BOMD with DFT	[178]
Li_12_Y cluster and its dimer	Characterize icosahedral superatom with high magnetic moment and preserved dimer integrity	BOMD with DFT	[179]
Pd 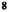 and Pd 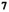 Au 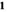 clusters	Au lowers melting point and promotes dimer diffusion	BOMD with DFT	[180]
CO 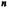 Pd 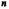 and Ni 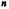 Pd 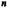 (*n* = 1‐10) clusters	BOMD used to initialize structures; reveals magnetic core‐shell behavior and spin localization on Co/Ni atoms	BOMD with DFT	[181]
WLi 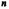 (*n* = 2‐12) clusters	BOMD confirms thermal stability of icosahedral W@Li 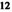 superatom with 18 valence electrons	BOMD with DFT	[182]
Pd 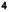 Ni 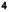 and Pd 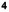 Cu 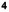 on defective graphene	BOMD‐based ADFT shows high interaction energies and small HOMO‐LUMO gaps on monovacancy and N‐doped graphene	BOMD	[183]

#### 2.2.2 Global Reaction Route Mapping

This iterative application enables systematic mapping of the PES, progressively revealing multiple minima and TSs. An influential example of this concept is the GRRM strategy introduced by S. Maeda, K. Ohno and K. Morokuma [29], which combines single‐ended methods, such as the SHS, with an automated exploration framework to systematically construct a reaction network. In this way, single‐ended approaches serve not only for the local determination of reaction pathways, but also as a global exploration tool that incrementally uncovers broader regions of the PES and their interconnections [30]. Single‐ended search methods are used to identify transition states (TSs) on PES using only a single stable molecular structure as input [184]. These methods aim to locate nearby saddle points and associated reaction pathways by iteratively modifying the initial structure in a physically motivated manner, without requiring explicit information about the product or reactant geometry structure. Abashkin and Russo were among the first to apply a geometrically constructed single‐ended method within first‐principles electronic structure theory [28]. Their approach was based on an earlier idea proposed by Dewar et al. [185], originally implemented in semiempirical frameworks. The key concept is to perform constrained energy minimizations on a hypersphere by reducing the tangential component of the gradient at each step. The endpoint of this constrained minimization corresponds to a point along the MEP connecting reactants and products. In this way, the method attempts to guide the system toward a transition state by following the topography of the PES starting from a known minimum. More recently, several advanced single‐ended search techniques have been developed. These include variants of the growing string method [186], the dimer method [187], and the single‐ended scaled hypersphere search (SHS) method [188, 189, 190]. These methods have enabled the identification of both transition states and local minima from a single starting structure, significantly broadening their utility in GO and mechanistic analysis.

**FIGURE 11:**
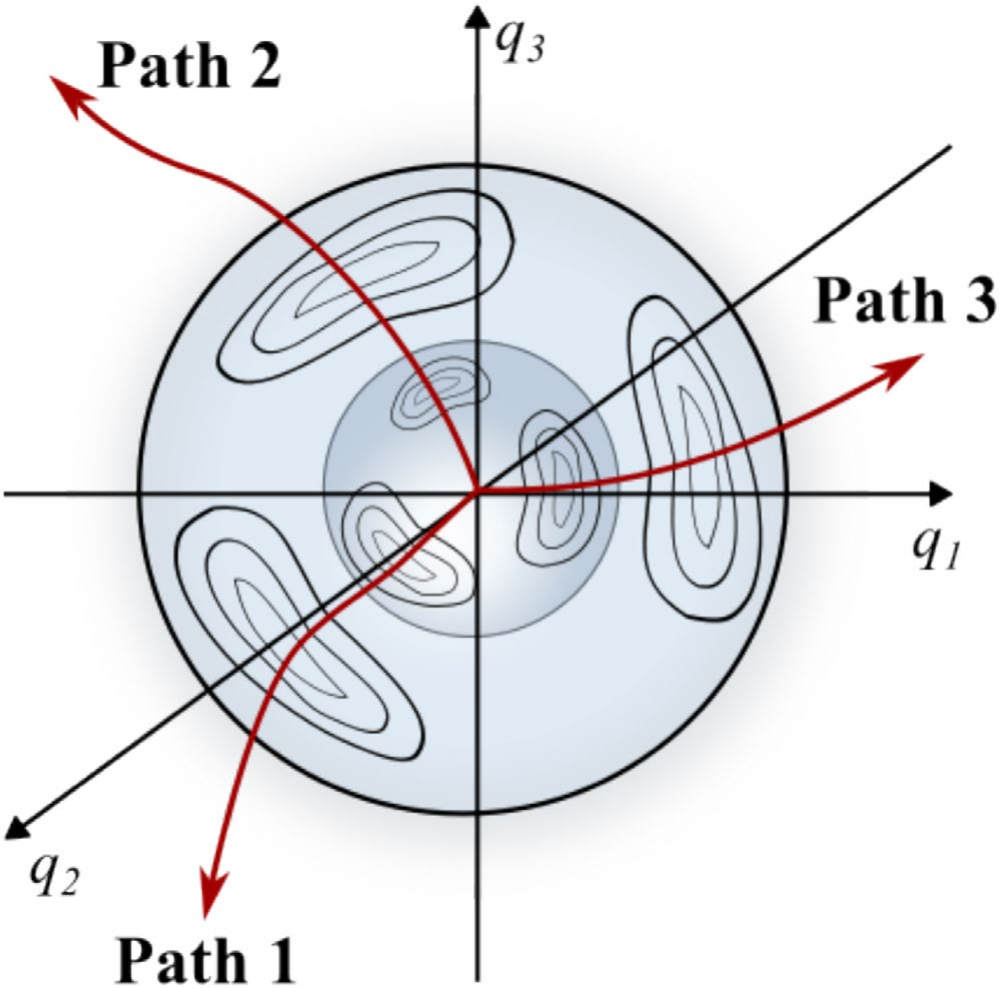
Conceptual diagram of the SHS method in a reduced normal coordinate space ($q_{1},q_{2},q_{3}$). See text for details.

**FIGURE 12:**
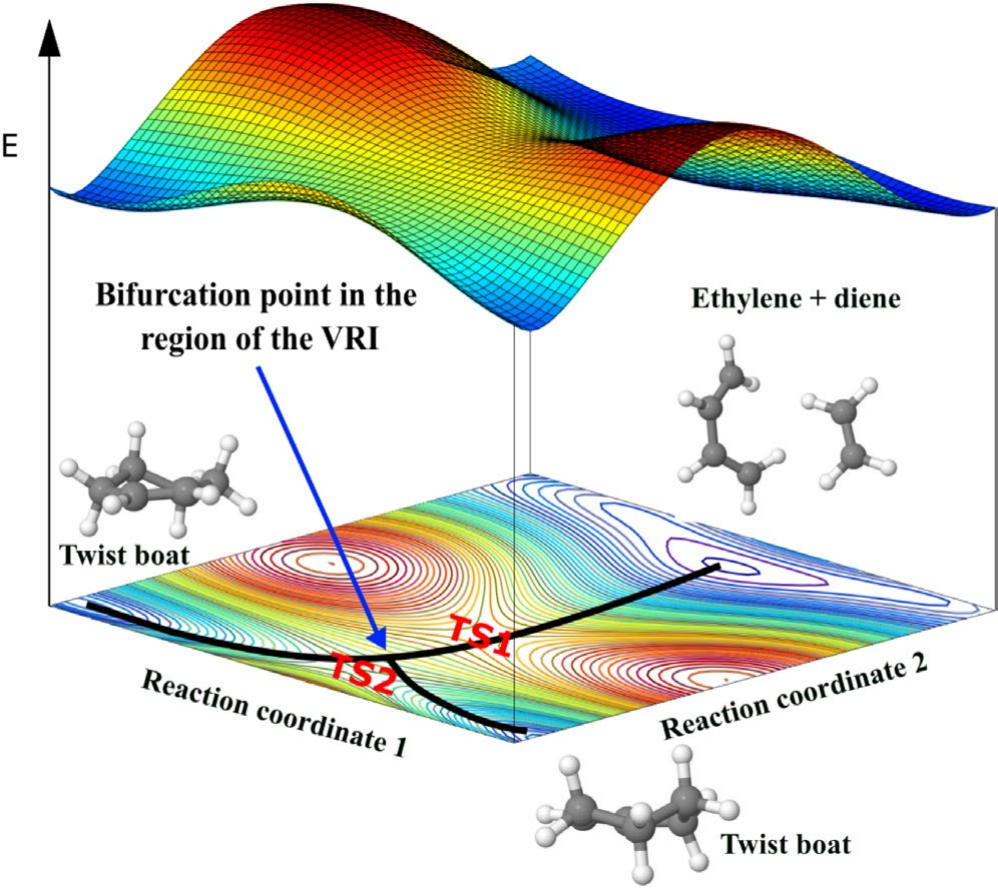
Model PES illustrating a valley‐ridge inflection (VRI) point and associated reaction pathway bifurcation. The surface depicts a reaction between ethylene and a diene, proceeding through two distinct transition states (TS1 and TS2) and leading to different twist‐boat product conformations.

In the SHS method, the system is described using scaled normal coordinates, $q_{i}$, where each mode is scaled by the square root of its associated vibrational eigenvalue at the initial minimum [189, 190]. The algorithm proceeds by taking steps in the radial direction of a hypersphere centered at the local minimum and performing energy minimization constrained to that hypersphere. By incrementally increasing the radius of the hypersphere, a sequence of configurations is generated that map the local topography of the PES. This procedure is repeated until a transition state is encountered. Once a TS is located, downhill pathways can be traced using the same hypersphere‐based approach, allowing for the identification of connected minima. In the SHS method, the system is described using scaled normal coordinates, $q_{i}$, where each mode is scaled by the square root of its associated vibrational eigenvalue at the initial minimum [189, 190]. The algorithm proceeds by taking steps in the radial direction of a hypersphere centered at the local minimum and performing energy minimization constrained to that hypersphere. By incrementally increasing the radius of the hypersphere, a sequence of configurations is generated that map the local topography of the PES. This procedure is repeated until a transition state is encountered. Once a TS is located, downhill pathways can be traced using the same hypersphere‐based approach, allowing for the identification of connected minima. Figure 11 provides a schematic illustration of this process. Recent extensions of the SHS framework have been proposed for more complex applications, including intrinsic reaction coordinate (IRC) calculations and automated TS localization in transition metal and organic systems [191]. Some PESs exhibit complex topologies, such as valley‐ridge inflection (VRI) points, which are commonly associated with symmetric reactions. As shown in Figure 12, this feature appears in the PES of a representative Diels‐Alder reaction [191]. The presence of VRI points can complicate the interpretation of reaction pathways, as they deviate from the conventional minimum energy path framework. To address such challenges, recent methodological developments aim to reinterpret and extend Dewar's original hypothesis within a more computationally rigorous and scalable framework [191]. These advances facilitate high‐throughput reaction discovery in increasingly complex chemical systems.

The SHS method and related single‐ended approaches have been applied across a wide range of chemical problems. Notable examples include global mapping of PESs for small molecules [192], mechanistic studies of reactions in molecular clusters [193], organometallic catalysis [193], and surface chemistry modeled using cluster‐based representations [194]. Table 8 presents additional representative examples [184, 186, 195, 196, 197, 198, 199, 200, 201, 202]. These methods continue to serve as important tools for exploring both thermodynamic and kinetic features of molecular systems.

**TABLE 8:** Set of ten representative applications of global reaction route mapping (GRRM) method in molecular systems.

System	GRRM contribution	GRRM method	References
Formaldehyde (${\text{H}}_{2}$CO)	Automated global mapping of minimum energy crossing points on S 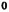 /S 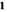 , S 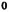 /T 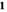 , and S 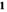 /T 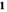 seams, identifying conical intersection structures relevant for photodissociation pathways	Anharmonic Downward Distortion following (ADD‐following) method applied to a two‐state penalty function	[195]
Small molecular reactions (A + B → X + Y)	Automated prediction of reaction mechanisms	Artificial Force Induced Reaction (AFIR) method	[196]
Solid‐solid phase transitions in periodic crystals	Discovery of reaction pathways and phase transition mechanisms involving both atomic and lattice degrees of freedom without predefined final states	Solid‐State Dimer (SSD) method	[197]
Photodissociation of small molecules (e.g., H 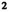 CO, NO 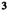 , HCOOH)	Automated exploration of adiabatic and nonadiabatic photodissociation pathways	Global reaction route mapping strategy	[198]
NH 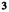 BH 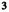 + (LiH) 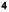	Discovery of 165 reaction pathways from single reactant input, without predefined products	Growing String Method (GSM)	[186]
TiN on Cu(111) surface	Elucidation of complex ALD mechanism involving H‐transfer and ligand exchange	Growing String Method	[184]
Various systems (organics, catalysis, photoreactions, surfaces)	Systematic exploration of complex chemical transformations and discovery of new synthetic routes	Artificial Force Induced Reaction	[199]
Formic acid on TiO 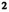 (101) surface	Exploration of temperature‐dependent decomposition pathways	Single‐component AFIR with RCMC navigation	[200]
Strecker and Passerini reactions (ab initio simulation)	Prediction of chemical reactions and yields through forward and backward kinetic simulations	AFIR combined	[201]
Pericyclic reactions (three test cases)	Identification of perturbation‐induced downhill bifurcations affecting reaction selectivity	Downhill bifurcation analysis based on intrinsic reaction coordinate topology	[202]

#### 2.2.3 Parallel Tempering Molecular Dynamics

PTMD, also known as replica‐exchange molecular dynamics (REMD), is an enhanced sampling technique designed to improve the exploration of complex PES by simulating multiple replicas of a molecular system at different temperatures [203]. This method is particularly effective for overcoming high‐energy barriers and improving configurational sampling, especially in systems with complex landscapes. In a PTMD simulation, N independent replicas of the system are propagated in parallel using MD, each at a distinct temperature. Low‐temperature replicas efficiently sample regions near local minima, while high‐temperature replicas enable the system to escape these minima by facilitating barrier crossing. At fixed intervals, exchange attempts are made between adjacent temperature replicas. These exchanges are evaluated using the Metropolis criterion [40], ensuring detailed balance and correct Boltzmann‐weighted ensemble statistics. Through these exchanges, configurations diffuse across temperature space, allowing the system to explore both high‐ and low‐energy regions more effectively.

**FIGURE 13:**
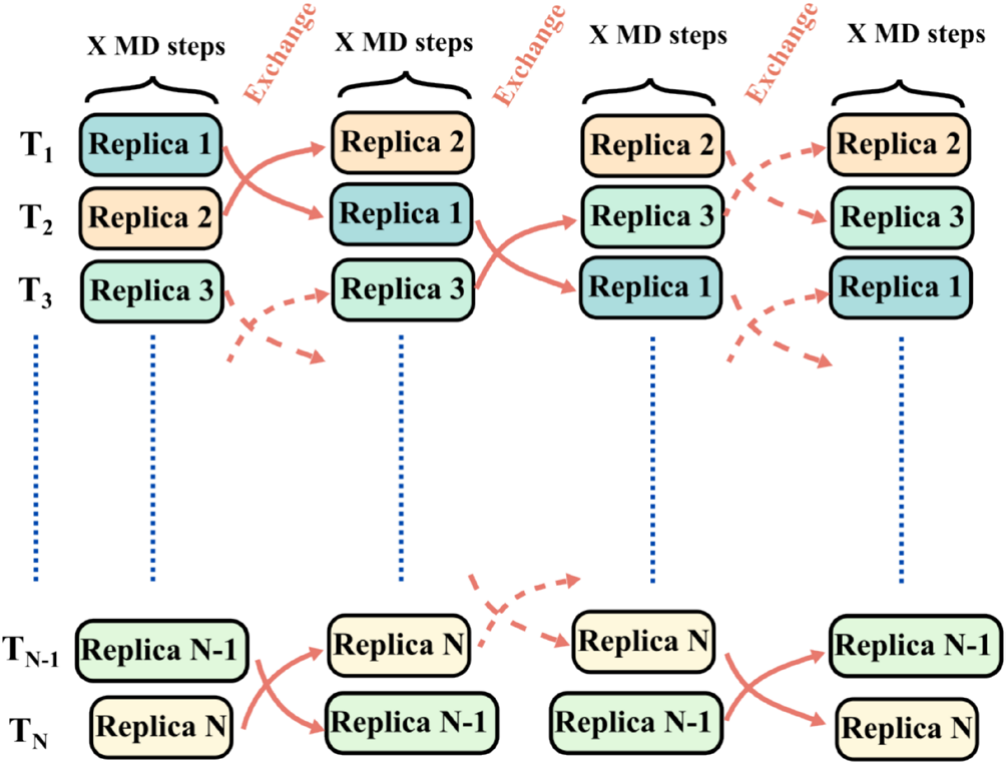
Schematic representation of the (PTMD). See text for details.

The efficiency of PTMD depends critically on the choice of temperature distribution. If temperature intervals are too large, exchange probabilities decrease, impeding communication between replicas. Conversely, overly small intervals increase computational cost without proportionate gains in sampling efficiency. Striking a balance between exchange rate and computational feasibility is therefore essential for optimal performance. PTMD has been implemented in electronic structure packages such as deMonNano and deMon2k [204], enabling first‐principles simulations across multiple temperature regimes [205]. Figure 13 illustrates the operational workflow of PTMD in the deMon2k code, including periodic exchanges of configurations between replicas. This implementation facilitates ab initio exploration of PESs, even for systems characterized by complex bonding patterns or multiple low‐energy conformers. The method has been successfully applied to water clusters such as the hexamer (H
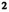
O)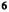 and octamer (H
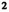
O)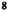, where accurate conformational sampling is essential for identifying global minima and understanding hydrogen bonding networks [205, 206]. Structures sampled during the simulation can be extracted from low‐ and intermediate‐temperature replicas and subsequently optimized to locate the most stable configurations. This approach captures not only kinetically trapped conformations but also thermodynamically relevant minima.

Table 9 summarizes representative applications of PTMD in chemical systems, illustrating its versatility in both GO and thermodynamic analysis [205, 207, 208, 209, 210, 211, 212, 213, 214, 215]. As computational resources and parallel architectures continue to evolve, PTMD is expected to remain a key methodology for high‐accuracy simulations across chemistry, materials science, and molecular biology.

**TABLE 9:** Set of ten representative applications of parallel tempering molecular dynamics (PTMD) method in molecular systems.

System	PTMD contribution	PTMD method	References
Au 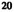 , Ag 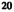 , Au 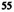 , Ag 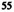 clusters	Correlation of melting behavior with isomer spectra using PTMD	PTMD with DFTB and multiple histogram method	[207]
${\text{SCN}}^{-}$(H 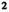 O) 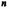 (*n* = 1 − 8)	Explored conformational populations and IR spectral shifts using PTMD	PTMD and thermochemical ensemble averaging	[208]
${\left({\text{Cl}}^{-}\right)}_{2}$ (${\text{H}}_{2}\text{O}$) 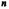 (*n* = 1 ‒ 6)	Located low‐energy isomers using PTMD	PT on empirical PES followed by DFT refinement	[209]
(H 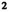 O) 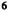	Demonstrated PTMD implementation in DFT (deMon2k) for simulating phase transitions in water hexamer	PTMD with localized basis set DFT	[205]
Au 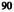 , Au 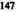 , Ag 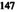 , Cu 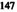	PTMD used to generate data for ML‐based structural landscape	PTMD combined with deep learning (convolutional autoencoders)	[210]
Cu nanocrystals (100‐200 atoms)	PTMD used to explore size and temperature dependent shape transitions	PTMD with DFT validation	[211]
Cu, Ag, and Au clusters (90‐201 atoms)	PTMD combined with harmonic superposition approximation (HSA) to study temperature‐dependent structural motifs and solid‐solid transformations	PTMD + HSA + DFT	[212]
CNO^–^(H 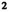 O) 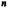 (*n* = 1‐8)	Conformational populations and heat capacities computed to assess structure and phase behavior	PTMD	[213]
Au nanoclusters on graphene and graphite	PTMD used to explore temperature‐dependent structures and diffusion behavior	PTMD + Wulff‐Kaischew + DFT‐based potential	[214]
(Bi 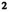 O 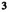 ) 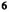 cluster	Identifies quasi‐degenerate global minima automatically	PTMD combined with Discrete Cosine Transformation	[215]

## 3 Machine Learning Drive Global Optimization

**FIGURE 14:**
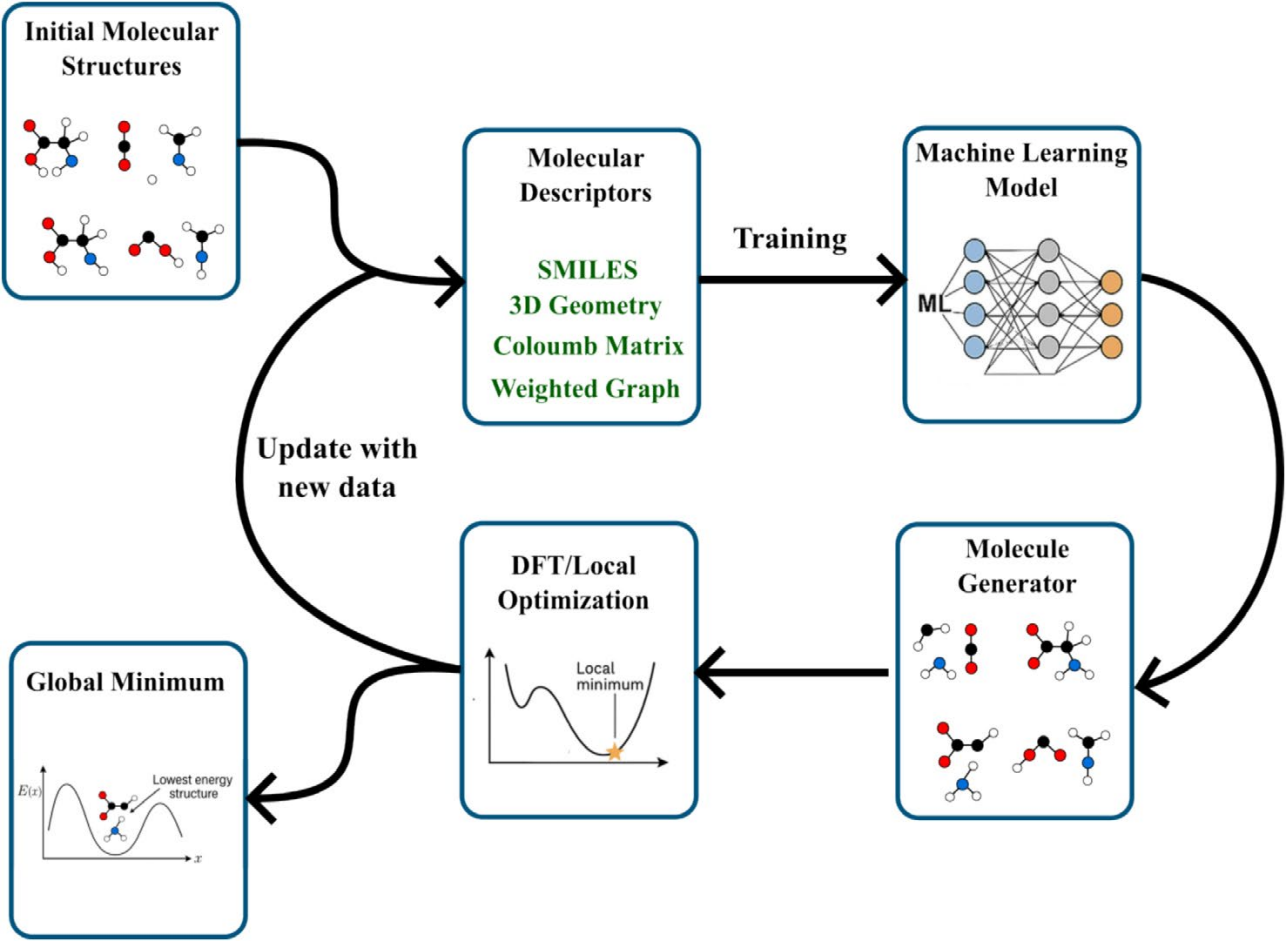
Schematic workflow of a ML drive global optimization framework for molecular structure prediction. See text for details.

It is important to point out to the reader that GO needs an incredible amount of energy and gradients calculations. This is why over the past two decades, considerable work has been done to advance GO methods. In particular, new approaches that offer faster computations, utilize ML and data‐driven materials‐science techniques [128]. In GO methods, ML models are trained on examples, meaning data points with known outcomes, so that they can later recognize similar patterns in new, unseen data. With sufficient training and an appropriate model architecture, the algorithm improves its ability to classify, predict, or generate results that align with human analysis or reference calculations. Although early applications of ML in chemistry date back to 1969 [216], the past two decades have seen rapid progress in using ML to predict molecular properties, especially energies and atomic forces [217, 218]. These models, typically trained on quantum mechanical datasets, can approximate the PES at a fraction of the cost of ab initio methods. As a result, ML has become a valuable tool for accelerating GO. Figure 14 illustrates a typical ML‐based workflow for molecular structure prediction. The process begins with a set of initial molecular structures, which are converted into numerical representations known as molecular descriptors. Common descriptors include SMILES strings [57], Coulomb matrices [219], three‐dimensional coordinates, and graph‐based encodings [220]. These representations are used to train a supervised ML model, such as a neural network or a kernel‐based regressor, which predicts properties like energy or stability.

Once trained, the model is used to generate new candidate structures by identifying configurations likely to have low energy [221, 222]. These structures are refined using local optimization techniques, often based on DFT, to obtain their minimum‐energy geometries. The refined results are then added to the training dataset, allowing the model to improve through retraining. This closed‐loop cycle, consisting of prediction, generation, refinement, and retraining, enables efficient exploration of chemical space. Rather than sampling all configurations exhaustively, the model learns to focus on the most promising regions of the energy landscape. As a result, ML‐based optimization can identify global minima more rapidly and with fewer quantum chemical calculations [218, 223]. ML approaches have been successfully applied to a wide range of systems, including molecular clusters, organic compounds, catalysts, and crystalline materials. These applications demonstrate the ability of ML to combine rapid prediction with accurate refinement, significantly accelerating molecular and materials discovery. Among the various ML strategies, neural networks (NNs) are widely used due to their flexibility and capacity to model complex, nonlinear relationships. However, ML also includes a broader family of techniques. For example, in supervised learning, active learning (AL) has emerged as an effective enhancement. AL techniques use the model's uncertainty to select the most informative new data points, thereby focusing computational resources on unexplored but chemically relevant regions of configuration space. This has proven especially valuable for surface adsorption studies, where evaluating all possible configurations is computationally infeasible [224].

Beyond NNs and AL, several other ML methods have been adapted for GO. Kernel‐based techniques, such as kernel ridge regression and Gaussian process regression (GPR), are particularly valued for their ability to quantify uncertainty, which supports efficient sampling through Bayesian optimization [225, 226, 227]. Probabilistic generative models, including variational autoencoders [228] and normalizing flows [229], can generate diverse candidate structures and explore low‐dimensional manifolds of the energy surface. Tree‐based methods, such as random forests [230] and gradient‐boosted decision trees [231], offer interpretable and computationally efficient approaches to property prediction and molecular screening. In parallel, graph‐based models such as graph neural networks (GNNs) [232] and message‐passing networks [233] have shown particular strength in learning molecular connectivity and structural motifs. Each of these techniques offers specific advantages in terms of predictive accuracy, sample efficiency, interpretability, and scalability. The optimal choice depends on the complexity of the chemical system and the goals of the optimization task. To illustrate their practical utility, Table 10 presents ten representative studies where ML has been applied to GM searches [227, 234, 235, 236, 237, 238, 239, 240, 241, 242]. Each entry highlights the system studied, the ML approach used, and its contribution to the optimization workflow.

**TABLE 10:** Set of ten representative example applications of machine learning (ML) drive global optimization in molecular systems.

System	ML contribution	ML method	References
Adsorbate‐surface (Fe 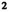 O 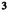 , TiO 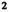 )	Accelerated SCF convergence and structure prediction	ML surrogates reduce DFT calls	[234]
Bulk oxides and surface reactions (e.g., TiO 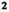 , ZnCrO 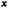 )	Construction of transferable NN potentials for global PES sampling	Neural Networks	[235]
Atomic clusters (Al 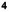 Si 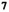 , 4Al@Si 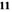 , Na_20_)	Accelerated GO via uncertainty driven exploration	AL with GPR and neural networks	[236]
Carbon clusters (C 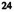 , C 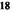 @Ir(111))	Accelerated structural search using surrogate energy models	On‐the‐fly GPR surrogate	[237]
Adsorbates on Rh(111) and Rh(211) surfaces	GO of adsorption geometries with reduced DFT evaluations	On‐the‐fly trained ML potential	[227]
Metal clusters (Pt 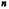 , n = 8‐14)	Accelerated GO via parameter transfer across cluster sizes	Deep neural networks with transfer learning (DNN‐TL)	[238]
Nanoclusters (Pd 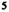 , Cu 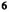 , Au 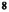 , Ni 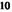 , Cu 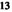 , Pd 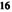 , Cu 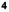 Au 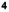 , Ni 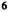 Pd 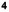 )	Accelerated GA‐based GO with reduced DFT evaluations	AL with ML potentials in GA framework	[239]
Ni‐CeO 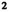 nanoparticles (Ce 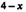 Ni 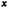 O 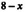 , x=1‐3)	GO of doped oxide nanoparticles with minimal DFT data	AL with regression + uncertainty estimation	[240]
Adsorbates on metal surfaces (e.g., CO/Pd(111), NH 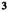 /Cu(100), CH 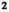 CO/Rh(211))	Accelerated GO of adsorption geometries with automated training	AL + moment tensor potentials (MTP)	[241]
Cerium oxide nanoclusters (Ce 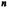 O 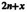 , n=2‐18)	Accelerated GO with near‐DFT accuracy across cluster sizes	AL + high‐dimensional neural network potential (HDNNP)	[242]

More recently, quantum computing has been explored as an extension of classical ML methods for GO. Quantum active learning (QAL) combines quantum machine learning (QML) models with uncertainty‐aware selection strategies to guide the identification of promising candidate structures. For example, the QMLMaterial agent framework employs quantum Gaussian process regression (QGPR) with fidelity‐aware and projected quantum kernels to explore energy landscapes of doped nanoparticles. In this workflow, structures with high predictive uncertainty are selected for evaluation using DFT or DFTB methods, and the resulting data are used to retrain the model. A recent application successfully identified the GM of a doped silicon nanoparticle (4Al@Si 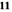), demonstrating the effectiveness of QAL for structure determination in complex systems [243]. QAL is especially promising for large systems because the computational cost of evaluating quantum kernels scales primarily with the number of training samples and only weakly with the number of qubits. However, the exponential growth of configurational space in such systems means that quantum chemical evaluations remain a major computational bottleneck. Despite this challenge, QAL offers a promising path toward data‐efficient GO by combining quantum‐enhanced regression with iterative model refinement.

## 4 Outlook

The search for global minima on molecular PES remains one of the most fundamental and challenging problems in computational chemistry. As reviewed in this work, a wide range of GO methods have been developed, broadly categorized as stochastic and deterministic approaches. Each method presents unique strengths and limitations, making their comparative analysis essential for selecting the most appropriate technique for a given chemical system. It would be desirable to account with data obtained under similar conditions (similar system type, similar system size, similar computational platforms, etc.), in order to be able to compare directly the methods considering various aspects like scalability, accuracy and computational cost, between the others. This comparison is outside the scope of this work. We hope this review will be inspiring for future work in this direction.

From our point of view among stochastic methods, GAs offer broad applicability and intuitive appeal due to their population‐based nature and biologically inspired operators. Their performance, however, strongly depends on the molecular representation used. When SMILES strings are employed, crossover and mutation often yield invalid molecules, greatly reducing efficiency. In contrast, the use of SELFIES encoding ensures chemical validity during genetic operations, dramatically improving robustness and computational yield. Nevertheless, even with robust encodings, GAs can be time‐consuming and may require extensive parameter tuning to converge in high‐dimensional landscapes. SA stands out for its conceptual simplicity and reliability in escaping local minima. Its gradual cooling scheme allows for effective sampling of complex PESs, especially in smaller or moderately complex systems. However, convergence can be slow, and the choice of cooling schedule is critical. SA is particularly effective in systems with many near‐degenerate conformers but less efficient in large or highly flexible chemical spaces due to its sequential nature. PSO is known for its parallelizable structure and good balance between exploration and exploitation. It performs well when gradient information is unavailable or unreliable, making it suitable for noisy or non‐differentiable PESs. However, PSO can suffer from premature convergence and swarm stagnation, particularly in complex or deceptive landscapes. BH has proven highly effective for molecular clusters and crystalline systems. By transforming the PES into a collection of local minima through repeated perturbation and local minimization, BH efficiently avoids high‐frequency modes and emphasizes basin connectivity. Its independence from gradient information and compatibility with diverse energy models are major advantages. However, it may become inefficient in high‐dimensional systems without additional biasing strategies or hybridization with ML techniques. The ABC algorithm mimics collective foraging and offers an elegant balance of diversification and intensification. Its major advantages include low parameter sensitivity and natural resistance to premature convergence. However, its global search capabilities may still require enhancement for extremely complex PESs, and it may not scale well to very large chemical systems. SSW is notable for its ability to capture both structural and kinetic information, thanks to its guided biasing and explicit sampling of transition paths. SSW is especially well‐suited for systems involving complex phase transitions or amorphous materials. Still, it requires careful tuning of bias parameters and can be computationally intensive without surrogate potentials. ML based methods have transformed GO by offering rapid approximations of the PES after initial training on quantum mechanical data. Their greatest strength lies in accelerating structure discovery while reducing the number of expensive ab initio evaluations. Nevertheless, ML methods are only as good as their training data. Poor generalization, high initial cost of data generation, and limitations in extrapolating beyond the training domain remain open challenges.

Among deterministic methods, PTMD is highly effective for systems with large energy barriers, particularly in hydrogen‐bonded clusters and disordered systems. Its capacity to simultaneously explore low‐ and high‐temperature configurations improves sampling completeness. However, it demands substantial computational resources, as multiple MD simulations must be run in parallel. Efficient temperature ladder design and high exchange frequency are key to successful application. MD provides quantum‐level accuracy and dynamic sampling of thermally accessible configurations. It is invaluable in studies of reaction mechanisms and transition metal complexes. However, its computational cost is high, and exploration efficiency can be limited, particularly in large systems. Techniques such as scaled‐mass dynamics and enhanced sampling methods partially mitigate these limitations. The SHS method represents a geometrically motivated approach for identifying transition states and local minima from a single starting structure. Its application to PES exploration in complex organometallic and surface systems is promising. However, the method is computationally demanding and highly sensitive to the choice of hypersphere radius, making it more appropriate for systems with a limited number of degrees of freedom. To address these limitations, a new constrained hypersphere minimization approach, referred to as the local coordinate method, has been recently developed in our laboratory [191]. This technique forms the basis of a novel single‐ended method that enables efficient exploration of complex PESs with reasonable computational cost and accuracy. The method is currently undergoing further development.

In summary, stochastic methods are generally more flexible and scalable to larger systems, though they may lack convergence guarantees and can be time‐consuming. Deterministic methods, by contrast, offer reproducibility and mathematical rigor but often suffer from poor scalability due to their unfavorable computational scaling. There is no one‐size‐fits‐all solution. Instead, method selection must account for the nature of the PES, the system size, the desired level of accuracy, and available computational resources. Looking forward, several avenues for improvement remain open. Hybrid approaches that combine deterministic guarantees with stochastic exploration, particularly those that incorporate ML potentials, offer a promising path. The integration of uncertainty quantification, adaptive biasing strategies, and more efficient energy estimators will further enhance search performance. Quantum‐enhanced methods and parallel computing architectures are expected to redefine the boundaries of feasible GO in chemistry. Finally, we would like to underline to the reader that the above statements reflect the authors' own point of view, developed after an extensive analysis of the cited literature has been performed.

## Conflicts of Interest

The authors declare no conflicts of interest.

## Data Availability

Data sharing not applicable to this article as no datasets were generated or analysed during the current study.
